# When no answer is better than a wrong answer: A causal perspective on batch effects

**DOI:** 10.1162/imag_a_00458

**Published:** 2025-01-29

**Authors:** Eric W. Bridgeford, Michael Powell, Gregory Kiar, Stephanie Noble, Jaewon Chung, Sambit Panda, Ross Lawrence, Ting Xu, Michael Milham, Brian Caffo, Joshua T. Vogelstein

**Affiliations:** Johns Hopkins University, Baltimore, MD, United States; Stanford University, Stanford, CA, United States; Child Mind Institute, New York, NY, United States; Yale University, Northeastern University, New Haven, CT, United States

**Keywords:** batch effects, mega-analysis, mega-study, harmonization, causal, connectomics

## Abstract

Batch effects, undesirable sources of variability across multiple experiments, present significant challenges for scientific and clinical discoveries. Batch effects can (i) produce spurious signals and/or (ii) obscure genuine signals, contributing to the ongoing reproducibility crisis. Because batch effects are typically modeled as classical statistical effects, they often cannot differentiate between sources of variability due to confounding biases, which may lead them to erroneously conclude batch effects are present (or not). We formalize batch effects as causal effects, and introduce algorithms leveraging causal machinery, to address these concerns. Simulations illustrate that when non-causal methods provide the wrong answer, our methods either produce more accurate answers or “no answer,” meaning they assert the data are inadequate to confidently conclude on the presence of a batch effect. Applying our causal methods to 27 neuroimaging datasets yields qualitatively similar results: in situations where it is unclear whether batch effects are present, non-causal methods confidently identify (or fail to identify) batch effects, whereas our causal methods assert that it is unclear whether there are batch effects or not. In instances where batch effects should be discernable, our techniques produce different results from prior art, each of which produce results more qualitatively similar to not applying any batch effect correction to the data at all. This work, therefore, provides a causal framework for understanding the potential capabilities and limitations of analysis of multi-site data.

## 1 Introduction

The 21^st^century has seen the advent of high-throughput techniques for acquiring data on an unprecedented scale. Collection of these datasets often occurs through consortia across different sites, requiring post-hoc data aggregation approaches. These “mega-studies” comprise numerous individual studies, rendering samples sizes substantially larger than any individual study and addressing the small-sample size woes associated with modern big biomedical data (Marek et al., 2022).

Unfortunately, aggregating data across diverse datasets introduces a source of undesirable variability known as a batch effect.Lazar et al. (2013)provide a recent consensus definition of batch effect: “the batch effect represents the systematic technical differences when samples are processed and measured in different batches and which are unrelated to any biological variation.” While these batch effects may not be immediately nefarious, their correlation with upstream biological variables can be problematic (Leek et al., 2010). When biological variables are correlated with batch-related variables, our ability to discern veridical from spurious signals is limited (Akey et al., 2007;Conrads et al., 2004). This problem has “led to serious concerns about the validity of the biological conclusions” (Leek et al., 2010) in data that may be corrupted by these biases; that is, it is unclear whether subsequent detected variability can be attributed to the biological variables, or to the so called batch effect. Unfortunately, the qualitative description provided byLazar et al. (2013)presents limited technical information about how batch effects can be detected or mitigated.

Many approaches model the batch collection or measurement process as a nuisance variable (Johnson et al., 2007;Leek & Peng, 2015;Leek et al., 2010;Pearl, 2010b;Pomponio et al., 2020;Wachinger et al., 2020;Yu et al., 2018). The implicit model justifying these approaches assumes batch effects are associational or conditional, but not causal. Such assumptions are strong, potentially unjustified, and often inappropriate. Two of the most prominent examples of these techniques are ComBat and Conditional ComBat (cComBat) (Johnson et al., 2007). These approaches have demonstrated empirical utility in various genomics and neuroimaging contexts (Pomponio et al., 2020;Zhang et al., 2020); however, it remains unclear when these approaches will be successful, and when they will fail. Specifically, it is unclear when they remove biofidelic variability or fail to remove nuisance variability (Bayer et al., 2022). We still do not know when they produce “wrong answers,” removing desirable signal or failing to remove spurious signal.

In this work, we develop a causal approach to define, detect, estimate, and mitigate batch effects. Our main conceptual advance is modeling batch effects as causal effects rather than associational or conditional effects. Given this structure, we introduce a formal definition of causal batch effects. This formal definition reveals the limitations of (typically inappropriate) assumptions implicit in existing approaches (Rosenbaum & Rubin, 1983,^1985^;Stuart, 2010) and provides a theoretical explanation for many limitations of batch harmonization noted inBayer et al. (2022).

Methodologically, we introduce a simple pre-processing strategy that one can apply to existing techniques for batch effect detection and mitigation. First, to detect and estimate batch effects, we introduce CausalcDcorr(Bridgeford et al., 2023;Wang et al., 2015)—building on modern, non-parametric statistical methods—to estimate and detect the presence of batch effects. Second, to mitigate batch effects, we introduce Matching cComBat—an augmentation of the ComBat procedure (Johnson et al., 2007)—to remove batch effects while limiting the removal of veridical biological variation. Our proposed techniques introduce the possibility of “no answer” to batch effect correction and detection; that is, the data are insufficient to make a conclusion either way.

We apply these methods to simulations and a large neuroimaging mega-study assembled by the Consortium for Reliability and Reproducibility (CoRR) (Zuo et al., 2014), consisting of more than1,700 individuals across27disparate studies. Our simulations and real data analysis demonstrate that existing strategies can, under many realistic use-cases, experience biases wherein they will confidently produce potentially erroneous conclusions regarding batch effects, where our proposed techniques either produce expected behaviors or avoid inference entirely. This work, therefore, represents a seminal effort to design methodologies which identify or overcome potential biases in the detection and correction of batch effects from multi-site data.

## 2 Methods

### 2.1 A conceptual illustration of the value of causal modeling of batch effects

Consider a simple example inFigure 1. We observen=300measurements in two batches, where one batch (orange) tends to sample younger people, and the other batch (blue) tends to sample older people. The observed outcome of interest is disease state (y-axis), and there is a single potential confounder, which is measured: age (*x-axis*). The solid lines indicate the true distribution governing the relationship between age and disease for both batches, which is unknown in practice. That the data-generating distributions differ indicates that a batch effect is present (red band). Techniques are desired to remove the batch effect given only the data measurements (outcome and covariate pairs, indicated as points). The two rows show two different settings: the top shows a case where the orange batch tends to be larger than the blue before any correction is applied (a positive batch effect), and the bottom shows the reverse (a negative batch effect).

**Fig. 1.**
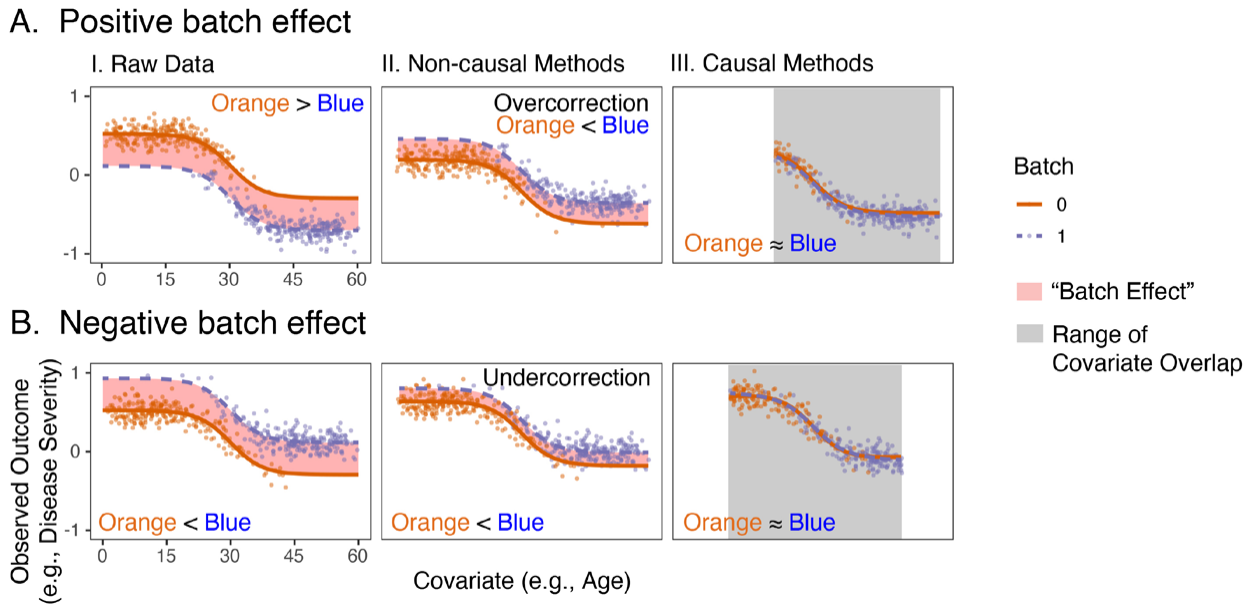
Non-causal batch effect mitigation procedures are subject to both over- and undercorrection and cannot rectify “confounding.” Our causally enriched methods address these issues. (I) shows the observed data (points), where color indicates batch. The orange line and the blue line indicate the expected outcome per batch, and the “batch effect” describes the observed difference (red band) between the expected outcomes. Ideally, after batch effect correction, the blue and orange lines should overlap. The orange batch tends to oversample people with younger ages, and the blue batch tends to oversample people with higher ages. (A) A scenario where the covariate distributions are moderately confounded and partially overlap, and the orange batch tends to see higher outcomes than the blue batch. (B) A scenario where the covariate distributions are moderately confounded and partially overlap, and the orange batch tends to see lower outcomes than the blue batch. (II) and (III) illustrate the corrected data after correction, via non-causal and causal methods, respectively. If the batch effect is removed, the orange and blue lines should be approximately equal. Non-causal methods attempt to adjust for the batch effect over the entire covariate range, and in so doing, are subject to strong confounding biases. Supposed “batch effect correction” instead introduces spurious artifacts (A) or fails to mitigate batch effects (B). Causal methods instead look to a reduced covariate range (gray box), finding points between the two datasets that are “similar,” and are not subject to these biases. Simulation settings are described inSupplementary Material D.2.

Figure 1Aillustrates an example with a moderate amount of covariate overlap, meaning that data from the two batches have similar (but not identical) age ranges. This problem is typically conceptualized via the location/scale (L/S) cComBat model ofJohnson et al. (2007), which can be generalized asPomponio et al. (2020):

$$Y_{ijk}\;=f_{k}\left(X_{ij}\right)+\gamma_{ik}\;+\delta_{ik}\epsilon_{ijk},$$

(1)

where the measurements$Y_{ijk}$for a samplejin batchiand dimensionkwith covariates$X_{ij}$are a linear combination of an overall “true” underlying property$f_{k}\left(X_{ij}\right)$with an additive batch effect$\gamma_{ik}$and a multiplicative batch effect$\delta_{ik}$for the error$\epsilon_{ijk}$, which is typically assumed to be normally distributed. This model is typically fit via regression, such as the cComBat procedure. Non-causal strategies such as cComBat learn from each batch, and then*extrapolate*trends across covariates (in this case, age) using the model to infer a relationship between the two batches. The problem is that this approach is strongly sensitive to the specifics of the extrapolation. Because the true data-generating distribution is unknown at the time of analysis, most non-causal approaches perform a linear extrapolation (Chen et al., 2022;Fortin et al., 2018;Johnson et al., 2007;Pomponio et al., 2020), where$f_{k}$is assumed to be a linear function. This is typically performed by “removing” the batch effect terms, for example, the “batch-effect corrected” measurements are

$$Y_{ijk}^{*}=\frac{Y_{ijk}-{\hat{f}}_{k}\left(X_{ij}\right)-{\hat{\gamma }}_{ik}}{{\hat{\delta }}_{ik}}+{\hat{f}}_{k}\left(X_{ij}\right).$$

When covariate overlap is imperfect, however, misspecification of$f_{k}$can be disastrous (Fig. 1A.II). While before correction the true data-generating distribution for the orange batch is higher than the blue batch, after correction, the blue batch is higher than the orange batch (the batch effect was*over-corrected*; i.e., too much correction was applied). Reversing the relationship between the blue and orange batches inFigure 1B, non-causal strategies are still unable to properly remove the batch effect and may still over- or undercorrect for batch effects. As a result, “batch-effect-corrected data” from non-causal strategies may not actually be corrected, and in many situations may be more different after “correction” than before. In other words, even though fundamental desiderata of batch effect correction are to decorrelate the relationship between the batch and upstream covariates of interest (Leek et al., 2010), the presence of such a correlation also hampers our ability to remove the batch effect.

However, inFigure 1A.IIIorFigure 1B.III, causal techniques focus instead on deriving conclusions within a range of covariate overlap in the data, where confounding is better controlled. The true data-generating distributions (and the points themselves) almost perfectly align after batch effect correction across the range of overlapping covariates, indicating that the batch effect has been removed, despite no foreknowledge of the true data-generating model.

In this manuscript, we posit that we desire that methods report confounding when it is present, something only causal methods do. Motivated by this perspective, we augment traditional approaches with causal machinery for batch effect detection and correction. Theory, simulations, and real data analysis demonstrate that traditional strategies to detect or correct batch effects from mega-studies lack the ability to identify confounding and, therefore, often add or remove batch effects inappropriately when covariate overlap is low. Therefore, such approaches cannot be trusted without further analysis. These issues highlight the primary challenges of performing valid statistical inference while pooling data across studies. This work, therefore, contributes to the ongoing effort to improve the validity and reliability of inferences in past and future mega-studies.

### 2.2 Models motivating different batch effect techniques

**Fig. 2.**
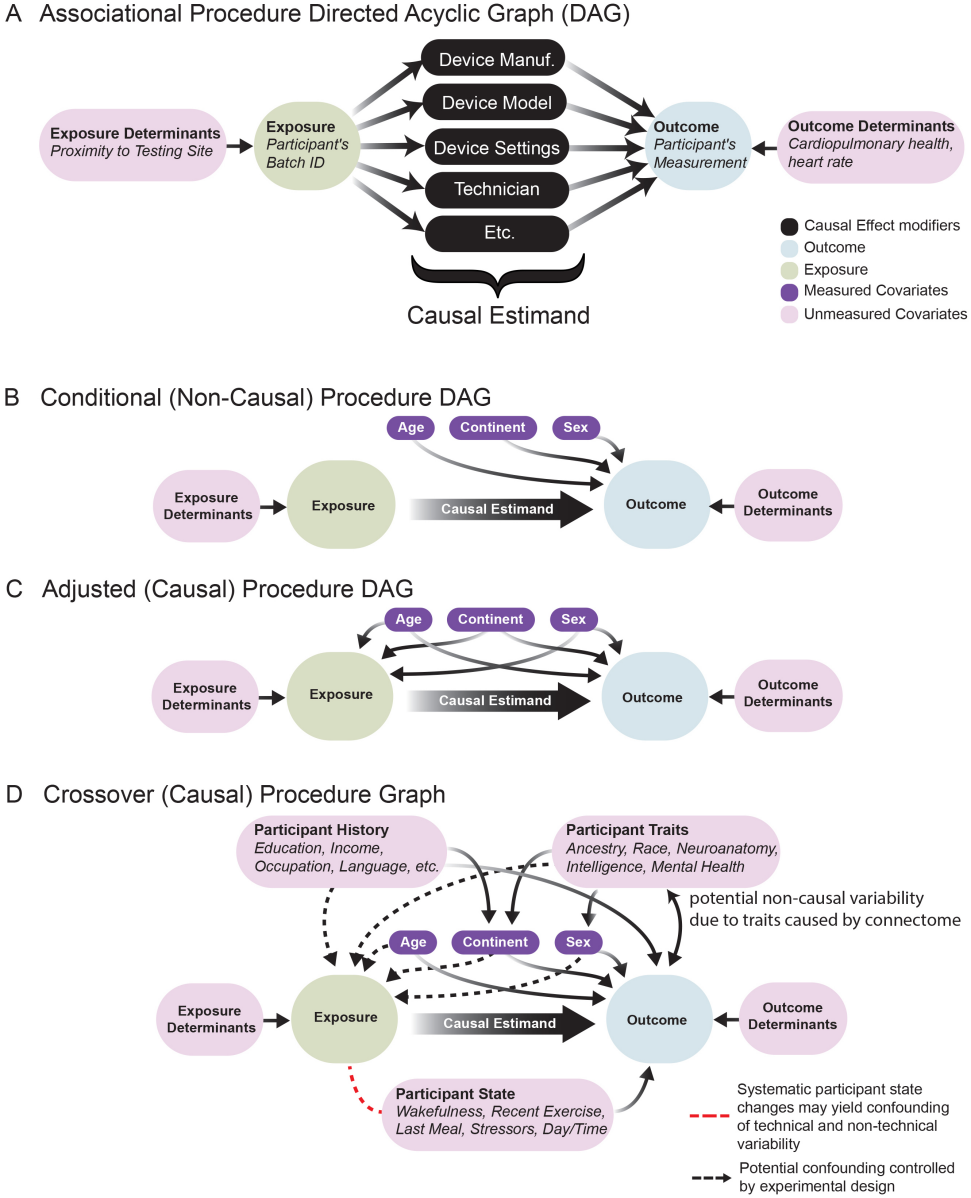
Causal Graph of Study Covariates. Causal graphs illustrating the underlying assumptions under which various procedures to detect or correct for batch effects are reasonable. Boxes represent variables of interest, and arrows indicate “cause and effect” relationships between variables. The causal estimand is the impact of the exposure (the batch) on the outcome (the participant’s measurement) and is a cumulative effect of effect modifiers (black variables) both known and unknown that yield batch-specific differences. The relationship between the exposure and the outcome is*confounded*if there are open backdoor paths (Pearl, 2009b). (A) Associational procedures and (B) conditional procedures are reasonable when there is no confounding. (C) Adjusted causal procedures are reasonable when backdoor paths can be blocked by measured covariates (Pearl, 1995,2009b). (D) Crossover procedures are reasonable under many forms of potential confounding, measured or unmeasured, so long as participant states are not changing or randomized. If participant states are changing or not randomized, the states must be measured (red arrow) to avoid aliasing batch effects with mediation effects due to participant state. Note that some participant traits (e.g., intelligence or mental health) may be caused by the connectome, and introduce the potential for bi-directional arrows in the causal graph with the measurement, due to a failure to reflect the underlying connectome free from measurement errors in the form of batch effects, visited inSection 4.

Here we build up the implicit modeling assumption underlying various approaches to mitigating batch effects. Our working example is that the exposure is batch, and the outcome is connectome. We want to know whether the differences between the batches are due to variability in the participants themselves who are measured, or veridical differences in outcomes across the two batches.Figure 2shows four different models and provides details of which kinds of variables are relevant in neuroimaging studies, though the concepts apply much more generally. Directionality of arrows (e.g.,T→Y) indicates that the variableTcan influence the variableY. Therefore, our goal is to identify a regime in which a strategy can provide evidence of a batch effect; that is, the degree to which the exposure influences the outcome (i.e., a*causal*effect).

We begin with the simplest approach, an “Associational” model (Fig. 2A). Using an associational model enables us to obtain valid answers to: “are differences in outcome associated with differences in exposure?” The answer to this question is often important, for example, in biomarker studies, but may be insufficient for determining causality. Dcorr(Székely et al., 2007) and naive ComBat (Johnson et al., 2007) (e.g., the model inEquation (1), but omitting covariate modeling via$f_{k}\left(X_{ij}\right)$) are methods designed to test and correct for associational effects. For example, if the batch and the outcome are dependent upon any covariates, which they often are in real studies, then an estimated associational effect is not a valid estimate of a batch effect (Fig. 2A). For instance, if the two batches differ because they have sampled individuals in different age groups, and the outcome is a function of age, then using this approach will lead to falsely concluding causal batch effects. However, if a batch effect is truly present, but somehow another covariate cancels out this variability, then this approach could falsely conclude there does not exist a batch effect when it is present.

The “Conditional” model (Fig. 2B) enables us to seek valid answers to a more nuanced question: “are differences in outcome associated with differences in exposure, after conditioning on measured covariates (Fig. 2B)?” If there are differences in outcome after conditioning, this can suggest that the differences are due to different exposures.cDcorr(Wang et al., 2015) and cComBat (Johnson et al., 2007) (e.g., the model inEquation (1)) are methods designed to test and correct for conditional effects. However, this model is subject to errors in the presence of confounding. The covariates might also impact the exposure, and if so, also impact our ability to identify a causal estimand, but without our knowledge. Imagine, for instance, that the two batches have covariates that partially overlap; for example, one sampled adolescents and adults, but not geriatrics, and the other sampled adults and geriatrics, but not adolescents. Conditional approaches (e.g., multivariate regressions, like cComBat) make implicit assumptions about how to interpolate outcomes across covariate distributions potentially unobserved in a given batch. When those assumptions do not reflect the real data, the results can be erroneous. In this light, the identified effect may be a veridical batch effect, or an artificial covariate effect, thereby leading to either false positives or false negatives.

The “Adjusted” model (Fig. 2C) enables us to mitigate this concern. Here, we seek valid answers to the following question: “conditioned on any impact of measured covariates on either exposure or outcome, do we see any residual differences in outcomes associated with differences in exposure?” This is effectively the model in most observational studies, which are a critical component of causal inference (Stuart, 2010). Our proposed CausalcDcorrand Matching cComBat (described below) are methods designed to test and correct for data following this model (Bridgeford et al., 2023). For example, to address the above concern, an adjusted model might discard all the adolescents and geriatrics, and simply focus on modeling the adults, for which we have data in both batches. While this reduces the sample size, it avoids the pitfalls of confidently concluding the presence or absence of a batch effect, even when it is not clear. Further, conclusions readily generalize to samples similar to the reduced samples, so many of the excluded samples may still be able to be batch effect corrected and used in subsequent inference. However, it also suffers from a potential confounding issue. While adjustment can be used to address confounding with measured covariates, it cannot be used to address confounding with*unobserved*covariates. Specifically, if there exist unobserved covariates that do not correlate with the observed ones and yet impact the outcomes, exposures, or both, then adjusted methods may lead to spurious results.

The “Cross-over” model (Fig. 2D) addresses this concern. In a cross-over investigation, each participant is measured across*all*exposure groups. When properly performed, cross-over models enable us to seek valid answers to questions such as “conditioned on almost all potential confounding, are differences in outcome associated with differences in exposure?” When the experimental design is a cross-over study and states are unchanging or randomized, simple ComBat approaches can be adequate, though the authors are not aware of any extensions of distance correlation or other non-parametric tests to these paired settings for high-dimensional data. If participant states change, then detected effects may confound both technical (i.e., due to scanner discrepancies) and non-technical (e.g., state-based effects, such as time of day) variability due to the changing participant states unless the order participants are measured in each batch is randomized or participant states are measured and suitably adjusted for, as per a suitable adjusted model.

The importance of these models can be formalized more rigorously through the statistical notions of causal consistency, positivity, and ignorability. Reasoning that data may satisfy these assumptions forms the cornerstone of causal inference from observational studies. In the context of an investigation with batch effects,*causal consistency*asserts that each individual has a set of potential measurements corresponding to each possible batch, and the observed measurement for an individual is equivalent to their potential outcome for the batch they were actually measured in (Cole & Frangakis, 2009). This criterion can be intuitively be reasoned for batch effects investigations, in that individuals could conceptually be measured across all batches (but we only observe their potential measurement for the batch in which they were measured).*Positivity*, equivalent to*covariate overlap*, states that for every combination of covariates, there must be a non-zero probability of being measured in any of the included batches (Rosenbaum & Rubin, 1983,^1985^).*Ignorability*asserts that given the observed covariates, the batch assignment and the potential outcomes are independent. Stated another way, conditional on the observed covariates, the batch assignment process is effectively random, and there are no unmeasured factors that influence*both*batch assignment and the potential outcomes (Rosenbaum & Rubin, 1983). While this may seem like an insurmountable hurdle, measured covariates are often sufficient conditioning sets so long as the measured covariates are suitably correlated with unmeasured variables (Pearl, 2009a). Together, these assumptions define when causal effects can, or cannot, be*identified*using the observed data. Causal methods such as matching or propensity weighting differ from more traditional methods in that they increase transparency for when these assumptions may be violated in the observed data. Additionally, these methods can aid in modifying the sample being analyzed such that causal conclusions regarding estimating and removing batch effects may be more reasonable in a reduced population.

Supplementary Material Aprovides rigorous definitions and mathematical models contextualizing the above definitions.

### 2.3 Detecting and mitigating batch effects

The causal graphs inFigure 2of batch effects make immediately clear their undesirability. If we want to learn about the impact of any upstream variable on the connectome via a proxy measurement,*batch effects may be a mediator of the relationship between that variable and the measurement*. If we want to learn about the impact of the connectome via a proxy measurement on any traits of a person (such as in a brain–behavioral investigation),*batch effects may introduce cycles in the causal graph, due to an inability to faithfully represent underlying neurological properties free from such measurement errors*. Both of these characteristics are immediately problematic for subsequently deriving causal conclusions, as failure to account for the batch effect may limit the identifiability of other potential estimands we may wish to subsequently investigate (Pearl, 2009b,2010a,^2014^). Removal of the batch effect on the data derivative represents a strategy to control for these problematic characteristics (via deletion of the arrow on the causal graph from batch to measurement), and motivates why studying, understanding, and deciphering strategies to detect or remove batch effects are desirable for subsequent inference tasks. Colloquially, this causal presentation delineates that batch effects are not just undesirable artifacts that could potentially yield spurious correlations (Leek et al., 2010), but also asserts that a failure to account for them*may prohibit principled subsequent inference*.

**Fig. 3.**
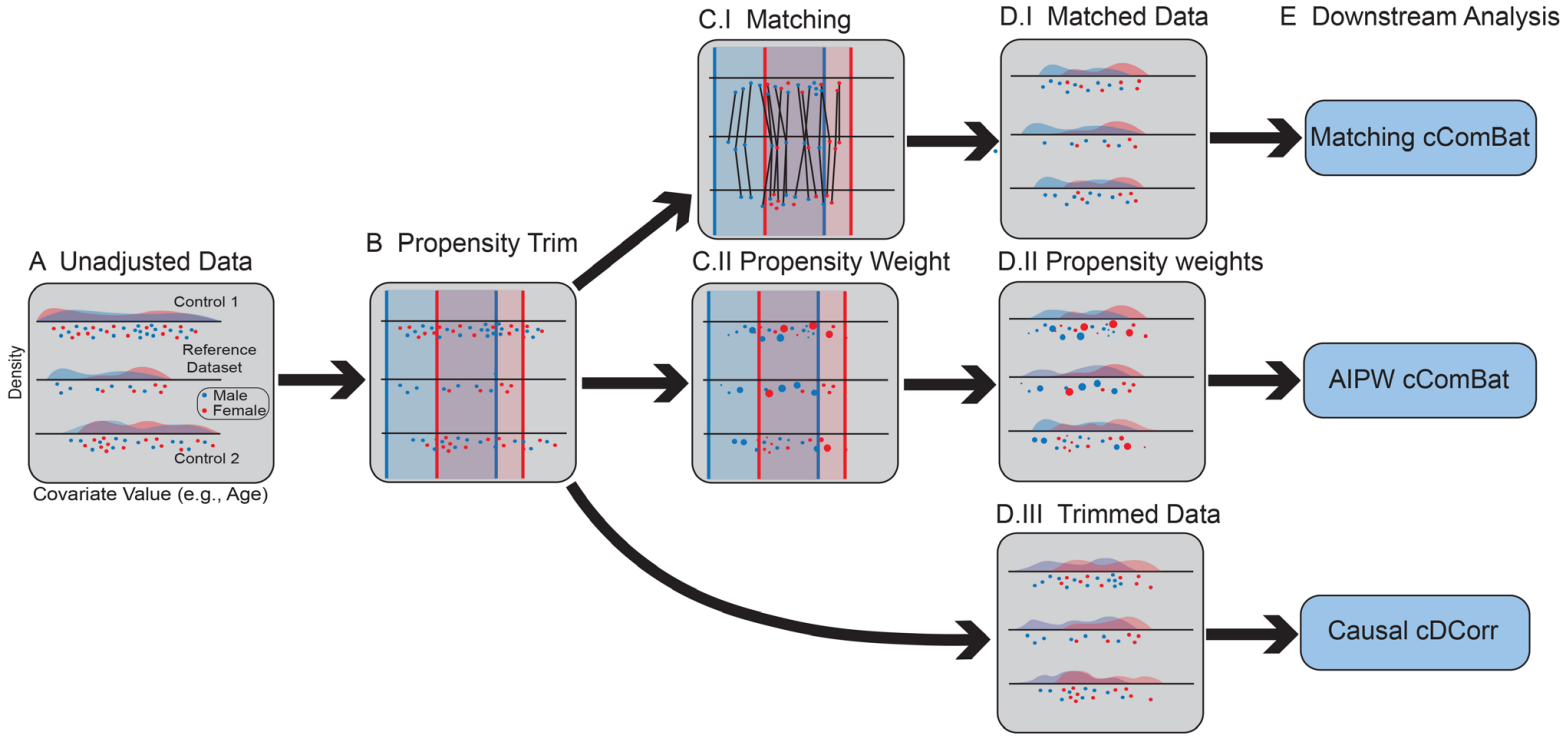
The demographic balancing procedure serves to demographically align poorly balanced datasets using causal approaches. (A) The unadjusted datasets are imbalanced in covariate distributions. The*reference dataset*is indicated. (B) Propensity trimming (shaded boxes) provides general alignment of demographics, such that no datasets will include demographics unrepresented in other datasets. (C.I) Samples from other datasets are matched to samples from the reference dataset and (D.I) samples without matches are discarded from subsequent analysis. The adjusted data after matching have nearly identical covariate distributions. (C.II) Samples are weighted according to their inverse propensities, such that samples which look non-representative of other datasets are down-weighted. (D.II) This can serve to also yield somewhat similar covariate distributions after re-weighting. (D.III) The trimmed data generally feature overlapping covariate distributions, but may not be identical across datasets. (E) Downstream analysis for batch effect correction or detection applied to the adjusted data (and potentially propensity weights) via Matching cComBat, AIPW cComBat, or CausalcDcorr.

At present, literature approaches for detecting and mitigating batch effects tend to (implicitly or explicitly) model the effects as associational or conditional, which often fail to adequately account for confounding biases. To this end, we propose a simple technique to augment classical strategies (Fig. 3; seeSupplementary Material Cfor methodological details):

1. Use classical causal procedures to re-weight the measurements from the batches so that measured covariate distributions across all batches are overlapping or balanced. For batch effect detection, we use vector matching (Lopez & Gutman, 2017), a form of propensity trimming which performs a multinomial regression of batch onto the covariates and then trims individuals with uncharacteristically low or high estimated probabilities (*propensities*) for any of the batches. For batch effect correction, Matching cComBat uses vector matching followed by a nearest neighbor matching (without replacement) of a “reference study” to the other studies in a mega-study. This matching may be performed many-to-one or one-to-many depending on the relative sizes of the reference study and the other studies. We propose the use of exact matching on categorical or binary covariates when possible, and Mahalanobis distance matching for continuous covariates. Our experiments and real data use-cases leverage a0.1distance caliper, which upper bounds the Mahalanobis distance between a given matched pair of individuals.
2. Apply traditional procedures for detection or correction of batch effects post re-weighting. We usecDcorrfor batch effect detection (Bridgeford et al., 2023;Wang et al., 2015), and cComBat for batch effect correction (Johnson et al., 2007;Leek & Peng, 2015;Leek et al., 2010;Pearl, 2010b;Pomponio et al., 2020;Wachinger et al., 2020;Yu et al., 2018).
3. Optionally, apply estimated batch effect corrections to excluded samples with covariates similar to the re-weighted samples or samples demographically dissimilar to the re-weighted samples. The former requires few additional assumptions, and may increase sample sizes for subsequent inference. Applying learned batch effects to demographically dissimilar individuals requires stringent assumptions regarding whether extrapolations to individuals demographically dissimilar from the re-weighted samples are appropriate.

Our proposed augmentations via classical causal procedures could be equivalently exchanged for other re-weighting procedures, such as inverse-probability weighting (IPW) or augmented inverse-probability weighting (AIPW). Our simulations explore an additional technique, AIPW cComBat, which uses augmented inverse-probability weighting to produce batch effect estimates. Through AIPW, we estimate propensity weights from the observed data, and incorporate these propensity weights into outcome model regression (J. Robins, 1986). While not the focus of this article, we explore the utility of these methods in simulation settings.

## 3 Results

### 3.1 Causal machinery helps mitigate limitations of traditional batch effect methods

**Fig. 4.**
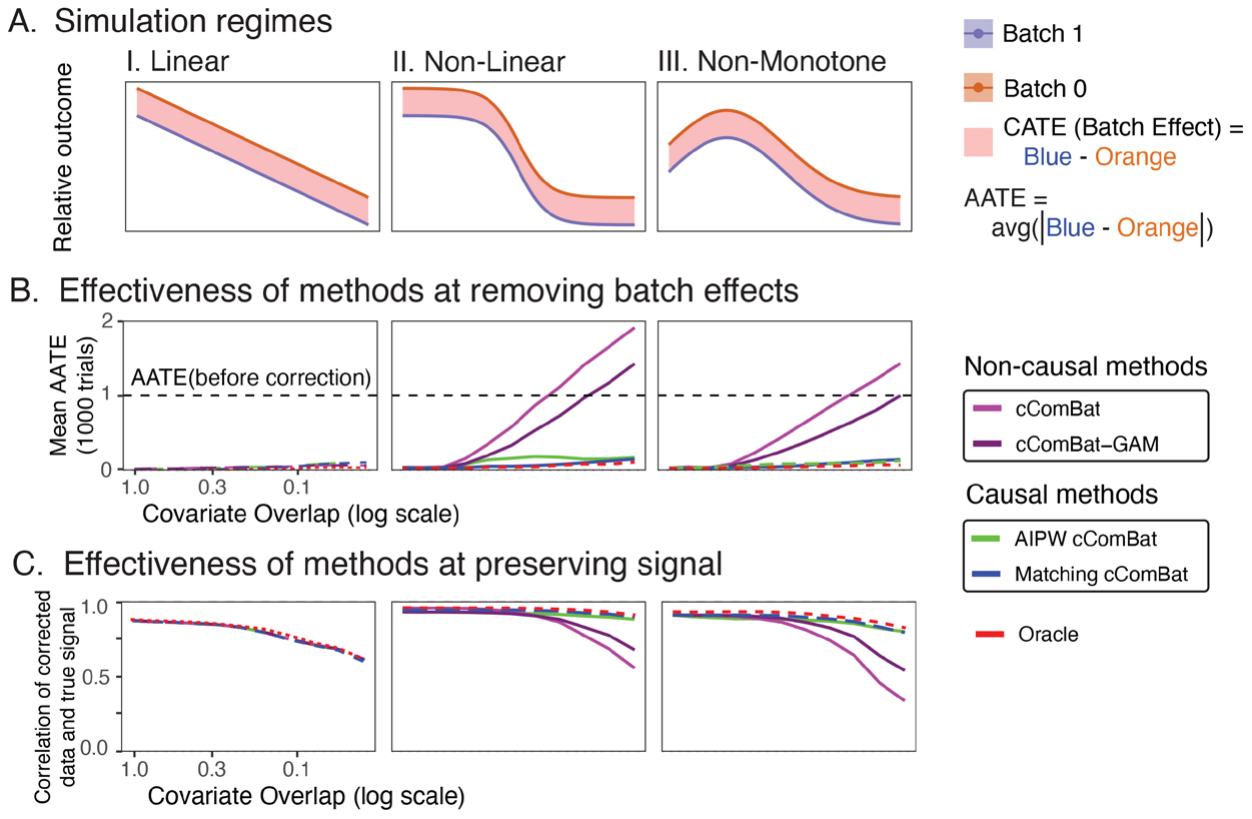
Simulation regimes illustrate that non-causal procedures are subject to strong biases without covariate matching. (A) illustrates the relationship between the relative expected outcome and the covariate value, for each batch (color), across (I.) linear, (II.) non-linear, and (III.) non-monotone regimes. The conditional average treatment effect (red box) highlights the batch effect for each covariate value. The average treatment effect (ATE) is the average width of this box, and the average absolute treatment effect (AATE) is the average absolute width of this box. In these simulations, the AATE before treatment is1. (B) The effectiveness of the techniques at removing the batch effect. Techniques with high performance will have a mean AATE after correction at or near0(the batch effect was eliminated). (C) illustrates the effectiveness of different batch effect correction techniques for preserving the underlying true signal. Techniques with high performance will have higher correlations with the underlying true signal. Simulation settings are described inSupplementary Material D.3.

Here we quantify the performance of causal and non-causal methods by generalizing the simulations fromFigure 1. In total,1,000samples are generated from one of two batches.Figure 4Ashows multiple simulation settings, including linear (left), nonlinear (center), and non-monotonic (right). Solid lines indicate the expected outcome at a given covariate value for a particular batch.Supplementary Material Dprovides details for the empirical settings of the simulations. The standard measure of effect size in the causal literature is called the “Average Treatment Effect” (ATE) (Rosenbaum & Rubin, 1983,^1985^). The red bands inFigure 4Ashow the treatment effect for all possible values of the covariate, so the ATE is just the average width of that bar, which happens to be−1here. In this case, we consider the Average Absolute Treatment Effect, which we denote by “AATE” for brevity, which is the average absolute width of the bar, which happens to be1here. Since the treatment here is batch, the goal is to remove the treatment effect, which requires correctly estimating the treatment effect and then removing it. WhenAATE=0(no batch effect), the covariate/outcome relationship is equal across the two batches, and non-zero otherwise.

We consider four different algorithms for this purpose: cComBat, cComBatGAM, Matching cComBat, and AIPW cComBat. These approaches are compared with the oracle, a batch effect correction technique which has prior knowledge of the true data generating model. We show the estimates of the mean AATE after correction over1,000trials for each method and each simulation setting as we vary the amount of covariate overlap (top row,Fig. 4B). Ideally, after batch effect correction is performed, the covariate/outcome relationship will be equal across the batches, and the AATE will be approximately zero. When the relationship between covariate and outcome is linear, all the methods work regardless of the amount of overlap (Fig. 4C.I). However, when the relationship is non-linear, the non-causal methods mis-estimate the batch effect unless there is a nearly perfect overlap, and the resulting data are qualitatively dissimilar across batches after correction, indicated by the mean AATE being far from0(Fig. 4B.II). In cases of extreme non-overlap, note that the data are similarly dissimilar after batch effect correction to before any correction was applied (AATE near or above the dotted black line). In contrast, Matching cComBat and AIPW cComBat correctly estimate and remove the batch effect, and demonstrate near optimal performance of the oracle. The results are qualitatively similar when the relationship is non-monotonic (Fig. 4B.III).

In addition to removing batch effects, it is critical that a batch effect correction technique preserves underlying signal in the data. We evaluate how well the corrected data reflect the true underlying relationship (linear, non-linear, or non-monotonic) between the covariate and the outcome. We compare the corrected data with the true relationship using Pearson’s correlation (Pearson, 1896), restricting to the matched samples so that the correlations are all computed with respect to the same set of sample inFigure 4C. Low correlations indicate that the data poorly reflect the true relationship, suggesting that regardless of whether or not there is a batch effect, the underlying signal has been perturbed. Again, regardless of the*a priori*presence of batch effects, causal methods tend to outperform non-causal methods for preserving the underlying signal in the data, and show performance near that of the oracle, particularly as covariate overlap declines.

The results are qualitatively and quantitatively similar when there is no batch effect*a priori*(the focus ofSupplementary Material D.5), indicating that non-causal methods can also introduce artifacts to the data when none is present. In contrast, causal methods show greater robustness to these situations too, with performance closely mirroring the oracle. Taken together, these results suggest the utility of causal methods for identifying and correcting for batch effects from data, as they have near-optimal performance in our simulation regimes for identifying and removing batch effects when batch effects are present, and avoiding the introduction of artifacts to the data when no batch effects are present*a priori*.

### 3.2 The CoRR Studies have disparate demographic characteristics

**Fig. 5.**
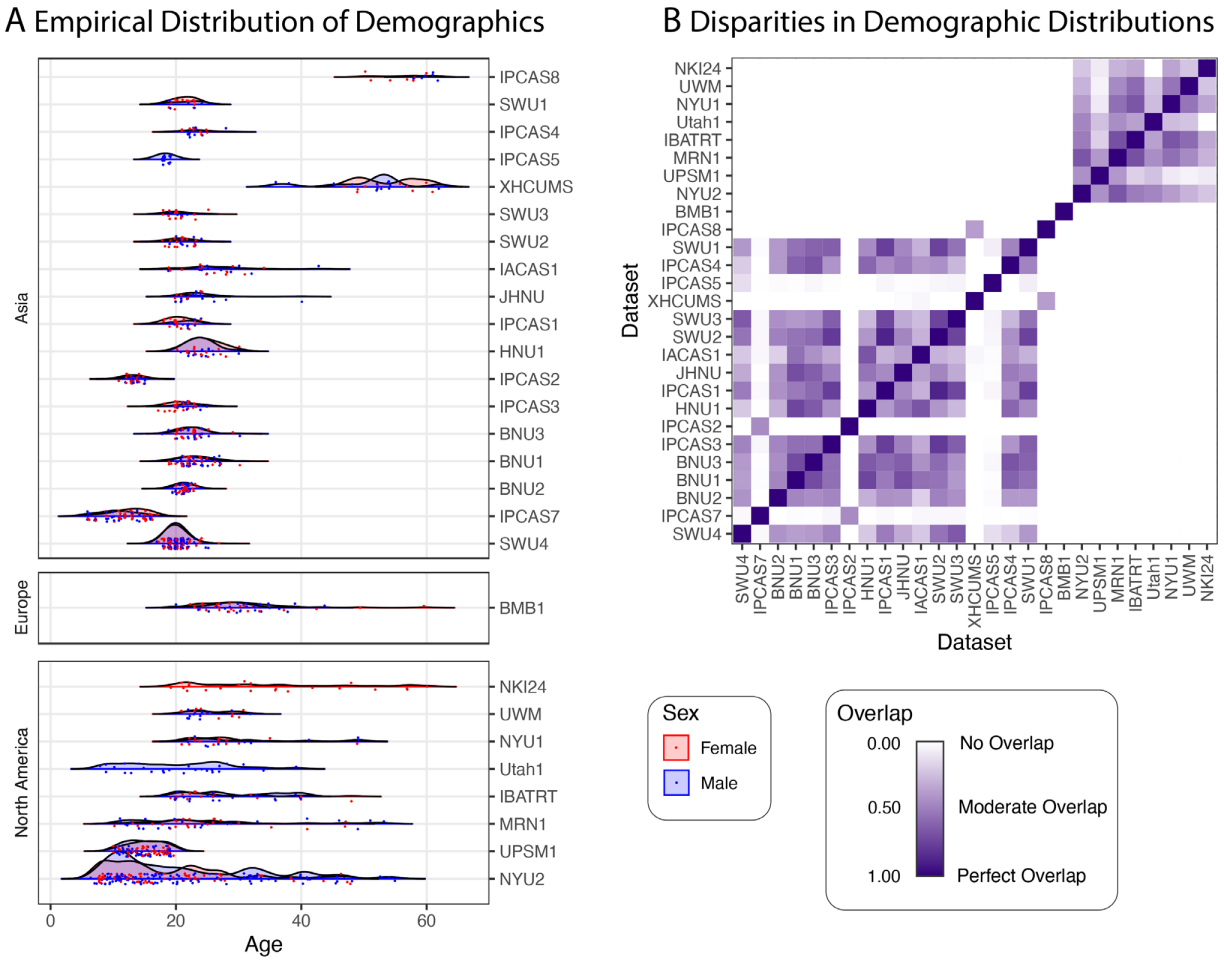
Demographic data for the27studies from the CoRR mega-study. (A) Each point represents the age of a participant corresponding to a single measurement. Rows are studies, boxes are continents, and color indicates sex.n=3,597samples are shown which featured age, sex, and continent information, and were successfully processed to connectomes. (B) Even with onlythreeobserved covariates (sex, age, and continent of measurement), the CoRR studies often show extremely limited covariate overlap (Pastore & Calcagnì, 2019). This makes inference regarding batch effects difficult.

The motivation for our work is the neuroimaging mega-study produced by the Consortium for Reliability and Reproducibility (Zuo et al., 2014), a collection of over3,500functional neuroimaging measurements from over1,700 individuals spanning27separate datasets. A full description of data pre-processing for the neuroimaging and covariate information is provided inSupplementary Material E.Figure 5Aexplores the demographic characteristics for the individuals in the CoRR mega-study. Many of the studies have a narrow age range, and several studies only include females. Because sex (Ingalhalikar et al., 2014;Satterthwaite et al., 2015;Weis et al., 2020), age (Hampson et al., 2012;Sala-Llonch et al., 2015;Varangis et al., 2019), and continent (as a surrogate for race and culture) (Ge et al., 2023;Misiura et al., 2020) are variables that have been associated with brain connectivity, they serve as measured demographic covariates used in our investigation.

Figure 5Billustrates the level of demographic overlap in the CoRR mega-study, using a distribution-free overlapping index (Pastore & Calcagnì, 2019) (seeSupplementary Material E.2for details). The CoRR mega-study includes many pairs of datasets with varying degrees of overlap, from high (near1) to low (near0). Further, many of the datasets do not overlap at all (overlap of0), making inference about batch effects impossible without making strong assumptions. This is particularly troublesome, as the covariate records for the CoRR mega-study common to all sites include only three covariates: age, sex, and continent of measurement. Additional measured covariates can only reduce the estimated overlap between the pairs of datasets, so having poor overlap on such a sparse set of covariates indicates that the actual demographic overlap is likely even lower.

### 3.3 Detecting Batch Effects in the CoRR mega-study

**Fig. 6.**
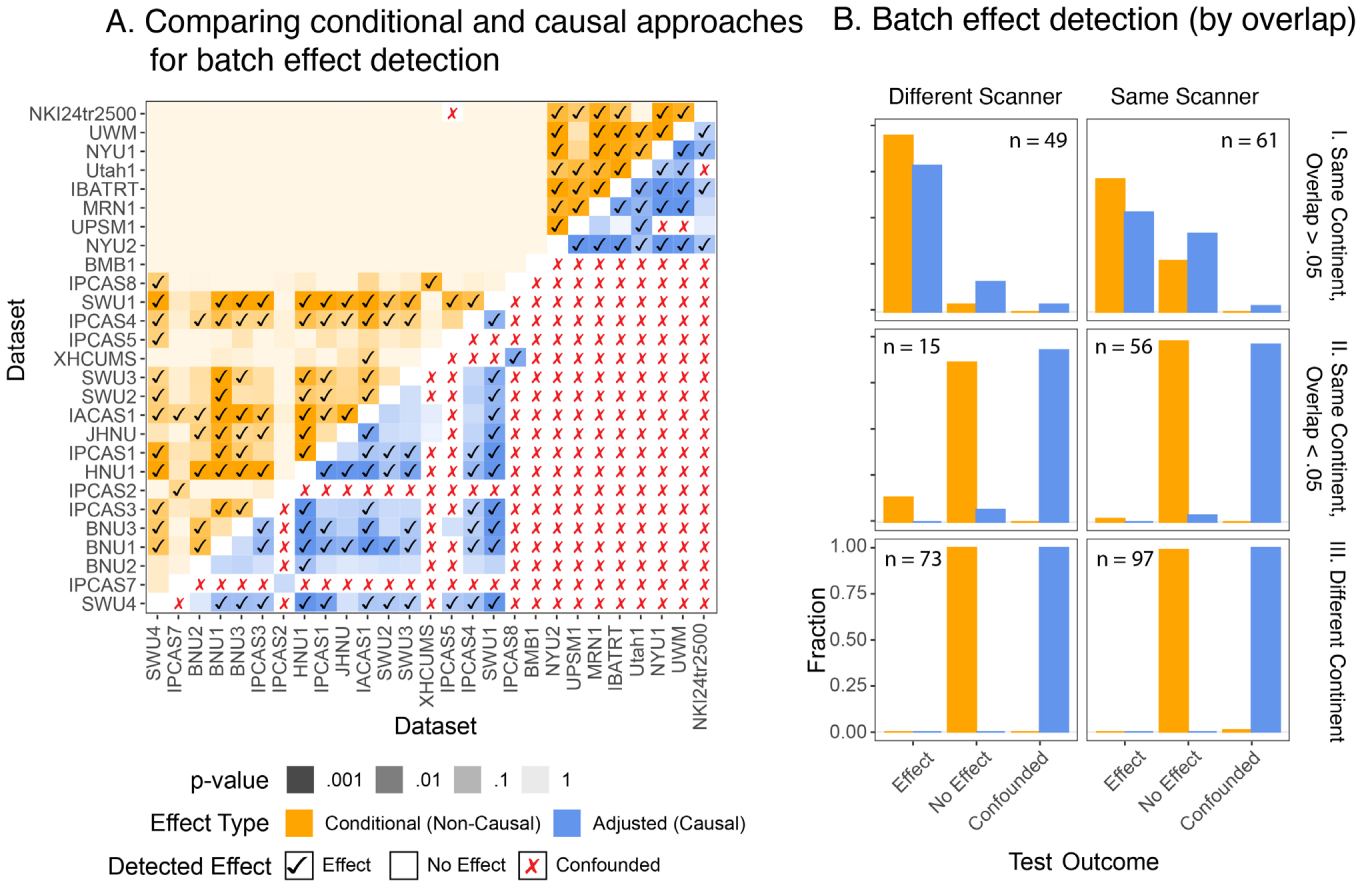
Comparison of types of effects between datasets from the CoRR study. (A) Heatmap of different types of effects (conditional and adjusted causal procedures) that can be used to detect differences between each pair of datasets in the CoRR study. Whereas most (25.8%) non-confounded conditional effects are not significant, most (66.1%) non-confounded adjusted effects are significant. (B).(I) Delineation of how one possible source of batch effects, scanner model, impacts significance rates of batch effects. Almost all pairs of studies conducted on different scanners with high covariate overlap (>.05) have discernable batch effects. The rate of detected effects is lower when the scanner model is the same. (B).(II) and (B).(III) When the level of estimated covariate overlap is lower (<.05) or zero (different continent), conditional effects never detect a difference across datasets. However, adjusted causal procedures instead report that the data are too confounded for subsequent inference and avoid running entirely.

Figure 6Afocuses on discerning the viability of different types of effects one could use to test for batch effects fromSection 2.2. For each pair of datasets in the CoRR study, we test whether (or not) a discernable effect is present, while controlling for age, sex, and continent differences between the individuals within each dataset. Intuitively, we would like to believe that if such a test rejects the null hypothesis in favor of the alternative, the data support that a batch effect is present. When we account for demographic covariates using conditional (non-causal) approaches (orange squares, top left), differences between the datasets are detected 26.9% of the time (checkboxes,cDcorr, BH correction,α=0.05). Conditional procedures may be “confounded” in extreme cases; for example, the batch effect conditional on age and sex is confounded between NKI24 and IPCAS5 because one dataset is entirely female and the other is entirely male, and the age distributions do not overlap (Fig. 5B). This results incDcorrbeing unable to compare the conditional distributions (across batch), since the conditional distributions are non-comparable across the batches in the data. Further, as explained inSection 2.2, the viability of these approaches as tests for batch effects is the assumption that the measured covariates are overlapping and non-confounding, which is overwhelmingly false for many of these comparisons, as shown inFigure 5B. Similar tests are performed using adjusted (causal) approaches inFigure 6A(blue squares, bottom right). Many pairs of studies could not have an adjusted (causal) effect estimated due to poor demographic alignment (242of351pairs of studies), where here “poor demographic alignment” corresponds to fewer than30samples retained after matching across the two sites. Notably, adjusted (causal) procedures can be used to estimate effects between all pairs of the American Clique (a group of separate studies consisting of similar demographics collected in North America, consisting of NYU1, NYU2, IBATRT, UWM, MRN1). After adjustment for the sample characteristics of the demographics associated with individual studies, a majority of adjusted (causal) effects (66.1%) remain significant (CausalcDcorr, BH correction,α=0.05).

Note that confounding (red “X”s) inFigure 6Afor causal effects extremely closely aligns with datasets with low covariate overlap inFigure 5B. A possible source of a batch effect could be different types of scanners were used to collect the data (Chen et al., 2022;Johnson et al., 2007;Pomponio et al., 2020), which is represented in our presentation as a causal effect modifier inFigure 2A. To this end, we aggregate the fraction of comparisons which report the indicated test outcome for different levels of covariate overlap (rows) when the scanner model is the same (right column) or different (left column). When the scanners are different and the covariates overlap, both causal and non-causal methods reliably detect batch effects a majority of the time, and they do so at a higher rate than when the scanner is the same (Fig. 6B.I). When the covariate overlap is low (Fig. 6B.II), or when the continent of measurement is different entirely (and the overlap is zero, (Fig. 6B.III), conditional (non-causal) procedures almost always fail to reject. However, adjusted (causal) procedures overwhelmingly report that a given pair of studies are demographically confounded, and are too different for any effect to be investigated.

Supplementary Material E.3computes numerous within-individual topological properties of functional connectomes, illustrating that ComBat-derived approaches do not tend to disrupt within-individual signal of the connectomes, which leaves open the possibility that while within-individual signal may be generally preserved, cross-individual variability may be altered by the techniques, and motivates our final analysis.

### 3.4 Traditional approaches produce disparate inference from techniques leveraging causality

**Fig. 7.**
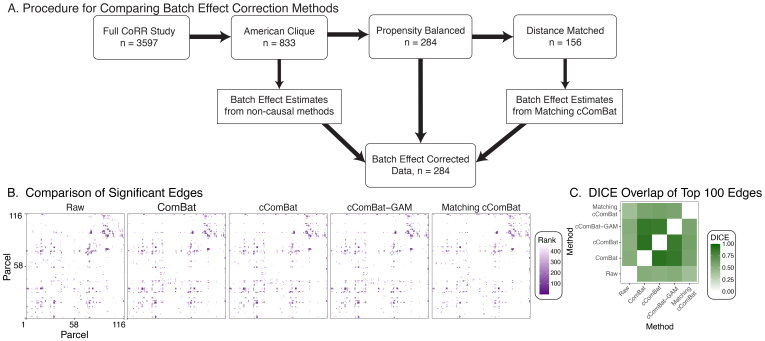
Significant Edges Before and After Batch Effect Removal. (A) The procedure for reducing the data to the approximately propensity balanced subset of the CoRR study. Non-causal methods learn and apply batch effect correction to the American Clique, which is further reduced to the approximately propensity balanced individuals. Causal methods learn batch effect corrections from the full matched data, and then apply the learned corrections to the approximately propensity balanced subset. (B) The presence of a sex effect (conditional on individual age) is investigated for each edge in the connectome. Significant edges are shown in rank order from largest (rank = 1) to smallest sex effect (α=.05, Benjamini Hochberg (Benjamini & Hochberg, 1995) correction). (C) the DICE overlap of the topn=100edges, by effect size, between all pairs in (B).

We investigate the strength of the sex effect, conditional on age, in the connectomes before and after batch effect correction (Fig. 7). Connectomes are filtered to the American Clique, a selection of datasets with overlapping demographics fromFigure 5A, and then either have no batch effect correction, ComBat, cComBat, or cComBatGAM (the non-causal approaches) applied to the resulting derivatives, and are finally filtered to an*overlapping subset*of individuals (Fig. 7A). Similarly, we apply Matching cComBat to the American Clique, which conceptually learns batch effect correction on the matched subset, and then applies the learned corrections to the overlapping subset. In this sense, while the manner in which the data were post-processed for batch effects differs, the actual individuals included and the techniques used for the subsequent analysis are identical. We test for a significant sex effect, conditional on age, using the generalized covariance measure. The generalized covariance measure (Shah & Peters, 2020) is a conditional independence test, which performs nonlinear regressions of the outcomes (connectivity) on the conditioning variable (age) and then tests for a vanishing covariance between the resulting residuals across sex. These tests can be adapted for two-sample testing regimes, such as for testing for differences across sex (Panda et al., 2025). The edges which show a significant sex effect conditional on age (generalized covariance measure, BH correction,α=.05) are colored from smallest test statistic to largest test statistic (rank = 1, dark purple), and edges without a significant conditional sex effect are in white (Fig. 7B).

While these plots appear superficially similar, we compare the DICE overlap (Dice, 1945;Sørensen, 1948) of the top100edges (by effect size) across all approaches. A high DICE overlap indicates that the subsets of edges are similar. ComBat, cComBat, and cComBatGAM tend to produce similar inferences. In contrast, they all have lower overlap with Matching cComBat (Fig. 7C), and all produce more similar subsequent inference to the raw connectomes (no batch effect correction) than Matching cComBat. This suggests that causal and non-causal strategies for batch effect correction can yield dissimilar subsequent inference, even though the selection of individuals being subsequently analyzed is the same.

## 4 Discussion

Succinctly, this manuscript is driven by two questions: (1) should data collected from mega-studies be combined or analyzed separately and (2) when it should be combined, the optimal strategies for doing so. Our primary conceptual contribution is establishing that batch effects can be formalized as causal effects. This formulation explicitly delineates the desirability of detecting and controlling for batch effects, as failure to do so limits the identifiability of other potential estimands of interest one may wish to study. Given this realization, we propose a framework for quantifying and removing batch effects in high-dimensional datasets featuring non-Euclidean measurements. We propose augmenting existing strategies for proper batch effect correction and removal by prepending causal balancing procedures.

We explore the advantages of these procedures by demonstrating that causal approaches (such as Matching cComBat, AIPW cComBat, and CausalcDcorr) yield empirically valid approaches to handling batch effects, or report that no inference can be made due to insufficient covariate overlap. This represents a principled approach over alternative strategies, which we show may introduce spurious biases and dramatically under- or over-estimate batch effects. We illustrate how, ignorant of potential confounding, otherwise principled strategies may misrepresent batch effects. This demonstrates that causal adjustment procedures can augment hypothesis testing by indicating when the data may be unsuitable to provide answers to a particular question due to irreconcilable confounding, rather than permitting false positives or false negatives.

Our neuroimaging use-case shows the utility of the methods developed herein for estimating and removing batch-related effects from large biological datasets. We demonstrate that many non-detected effects in the CoRR study may yield false conclusions due to excessive confounding between datasets. When demographic overlap was high and the scanner differed, batch effects were nearly always detectable using conditional (non-causal) or adjusted (causal) procedures. Conversely, with lower demographic overlap, effects were entirely undetectable by conditional procedures. Adjusted (causal) procedures explicitly delineate when the datasets are overly confounded by demographic covariates. We believe this to be a more desirable conclusion, as it highlights that in many cases, even with infinite data following similar covariate distributions, determining the presence or absence of a batch effect would be impossible due to high demographic confounding.

We conclude that batch effect correction represents a theoretically appropriate step supported by simulations for mega-studies with sufficient covariate overlap. When such overlap exists, causal methods demonstrate substantial performance improvements over alternative methods for eliminating undesirable spurious variability without distorting underlying veridical signal. Our methods identify different downstream demographic effects compared with prior approaches, even though the same individuals were analyzed, whereas prior approaches produce conclusions that are qualitatively more similar to the unharmonized data than causal approaches. These approaches are established in the causal literature to permit numerous false positives and false negatives (Ding & Miratrix, 2015;Forstmeier et al., 2017). Together, our work rigorously defines and studies measurement bias and strategies to mitigate demographic confounding in complex, non-Euclidean data.

### 4.1 Limitations

Researchers may perceive a tradeoff: when datasets lack high demographic overlap, model limitations can impart substantial bias on the investigation’s conclusions. Conversely, enforcing demographic overlap through propensity trimming or matching may seem to yield fewer samples or narrow the scope of conclusions. In practice, with biological data lacking ground truths, we cannot evaluate the introduction of imparted residual bias or determine whether downstream conclusions stem from these biases.

We do not claim that our specific choices for re-weighting measurements are “optimal.” Rather, we chose simple, principled approaches for illustrative purposes. Our proposed methods can be conceptualized as “bias-corrected” matching approaches, where we combine matching approaches as a non-parametric data pre-processing step with subsequent regression adjustments (Abadie & Imbens, 2011;Rubin & Thomas, 2000). These methods have been shown to reduce model dependence in parametric settings, such as those leveraged by cComBat or cComBatGAM (Ho et al., 2007). Our simulations elucidate that even these simple balancing approaches sufficiently highlight substantial shortcomings in existing approaches for detecting or correcting batch effects, and that causal machinery may mitigate these shortcomings.

In our real data analysis, we used only three covariates: age, sex, and continent of measurement (as a surrogate for race)—these being the only covariates collected across the CoRR study. While we acknowledge this limited covariate set is likely insufficient for formal harmonized analyses, it serves our exploratory purpose of illustrating the differences between causal and non-causal analyses. Moreover, even in mega-studies that collect more comprehensive covariate sets, many analyses focus primarily on age and sex for harmonization. We believe insufficient evidence exists to support the adequacy of such limited covariate sets for harmonization, and our work demonstrates how this limitation may lead to problematic confounding biases.

It is likely that other methods, such as extensions of our proposed AIPW cComBat, may be more appropriate in certain contexts than matching-based methods. In particular, for matching-based methods, one would typically restrict the study population (across all studies) to the “narrowest” covariate range, for example, the “intersection” of the covariate distributions across the included studies. AIPW-based methods may instead present promise for aligning each dataset to a broader “reference,” and then sequentially estimating batch effects for each non-reference group against the broad reference group. This may be complementary to current efforts to produce so-called lifespan reference curves (Zhu et al., 2024), which propose harmonizing datasets on the basis of reference templates gradually developed over time. Our work highlights that the appropriateness of such comparisons requires careful consideration of the demographic and phenotypic characteristics of both the groups used to develop these reference templates as well as the new datasets being aligned against the reference, which we believe has been insufficiently explored by the current literature. Further, AIPW-based methods generally offer the “doubly robust” property in parametric settings, where subsequent inference may be consistent if either the outcome or the propensity score model is correctly specified, affording two opportunities for correct estimation (J. M. Robins et al., 1994). Of note, our proof-of-concept AIPW cComBat implementation outperforms cComBat and cComBatGAM approaches in simulation environments. However, these methods are more difficult to adapt to present approaches due to the fact that they necessitate the incorporation of weighting schemes to batch effect correction techniques. It is possible that there are other desirable characteristics (such as generalizability to broader target populations) in which AIPW-based methods are more optimal than matching-based methods, or alternative causal approaches that are more performant than those investigated here.

Our work highlights the question of how to properly interpret multi-site scientific studies, particularly regarding internal and external validity (Degtiar & Rose, 2023;Patino & Ferreira, 2018). When we re-weight data to impose demographic overlap, we can make internally valid conclusions in regions of the covariate space that support analysis, albeit potentially for a narrower set of covariate values. Our procedures learn a batch effect correction for a matched subset of individuals, with conclusions methodologically valid for individuals within a range of covariate overlap with this re-weighted population. This approach allows investigators to benefit from causal methods while minimizing discarded samples, balancing internal validity with statistical power for subsequent investigations. Less conservative approaches might attempt to apply the internally valid batch effect correction to a wider target population, requiring potentially dubious extrapolations of estimated batch effects to new covariate values. In such contexts, a more conservative approach would be to analyze data separately and derive conclusions across subsequent analyses (i.e., via meta-study).

While causal re-weighting procedures will attempt to impose demographic overlap (when possible), we explicitly avoid making recommendations as to sample sizes sufficient for subsequent inference. Rather, we believe that these balancing procedures should be considered in tandem with the proposed inference question (e.g., after correcting for batch effects), and subsequent conclusions described in terms of the retained sample. For instance, if one wishes to learn a relationship between age and brain connectivity across the lifespan from multiple datasets, but a “matched sample” only includes individuals between20and25across the different datasets, perhaps a meta-study would be more appropriate than a mega-study incorporating batch effect correction techniques. If one wished to continue with a mega-study approach, care should be taken that subsequent inference methods are sensitive to sample sizes (e.g., at a fixed effect size, a method would provide less confident answers with fewer samples) and that the inference is presented alongside descriptors of the retained sample (e.g., explicitly delineating that the analysis only applies to individuals between20and25). Our software implementations, and consequently our simulations and real data analyses, default to raise errors if fewer than30samples are retained, a typical threshold for central limit theorem-based inference (such as regressions) (Rice, 2006), but this should not be used as a strict criterion.

Another limitation is that our methods exchange traditional statistical assumptions for sets of causal assumptions. This allows us to delineate a relatively understandable context (intuited via causal graphs and notions of covariate overlap) in which traditional batch effect correction techniques may be valid, and illustrate that data from mega-studies often do not resemble these assumptions. Even if “all models are wrong” (Box, 1976), their utility presupposes they loosely capture elements of the real data, which we show is frequently not the case for mega-studies.

### 4.2 Future work

This work suggests that harmonized analyses should be conducted with both harmonized measurement parameters and demographic distributions, departing from current practices in many mega-studies (Di Martino et al., 2014,^2017^;Yamashita et al., 2019;Zuo et al., 2014). Post-hoc batch effect detection and removal present theoretical and conceptual inconsistencies regarding internal validity when not viewed through a causal lens. This is evident in the poor demographic overlap in popular neuroimaging studies such as CoRR and ABIDE (Di Martino et al., 2014,^2017^). While SRBPS (Yamashita et al., 2019) shows greater demographic overlap, both ABIDE and SRBPS introduce additional complexities by using neurodivergences in participant recruitment. Neurodivergent brain features may cause symptoms of different neurocognitive behavioral phenotypes (Hashem et al., 2020;Konrad & Eickhoff, 2010;Vogelstein et al., 2019). If these phenotypes are then utilized in subsequent participant recruitment, this could potentially introduce unusual biases to the problem of identifying the batch effect due to selection biases or differential-exposure measurement errors (Bareinboim & Pearl, 2012;Nebel et al., 2022;Pearl, 2014). This can be conceptualized as participant neurocognitive phenotypes yielding violations of the ignorability assumption (Bridgeford et al., 2023) and rendering some causal effects unidentifiable (Pearl, 1995,2010a). While this problem has been defined for data fusion (Bareinboim & Pearl, 2016), its impact on batch effect detection or correction in high-dimensional biological studies remains unclear.

Recent work has proposed the use of deep learning methods for harmonization of magnetic resonance data derivatives such as T1w and T2w anatomical images (Hu et al., 2024;Liu & Yap, 2024). While to our knowledge no such developments have been proposed for fMRI, dMRI, or derivatives thereof (such as connectomes), we believe that these will likely be on the horizon. The methods proposed are heavily complementary to deep learning methods. In particular, deep learning methods are flexible for learning complicated batch effects from the data, and may hold similar promise to traveling subject datasets for estimating and removing batch effects (Liu & Yap, 2024). However, deep learning methods are known to be vulnerable to data dissimilar from that previously seen, known as the dataset or distribution shift problem, across many domains (Arjovsky, 2021;Ovadia et al., 2019;Taori et al., 2020). This is a particular concern for multi-site mega-studies, where as we have illustrated demographic overlap cannot be anticipated. We, therefore, do not anticipate that deep learning-based batch effect correction methods will be immune to these concerns. We believe that incorporating many of these techniques with approaches such as vertex matching or other causal balancing procedures, as we propose inSection 2.2, may represent a future area of interest.

Future work will focus on applying these methods to correct batch effects in mega-studies such as the Adolescent Brain Cognitive Development (ABCD) (Karcher & Barch, 2021), which includesN>11,000demographically diverse individuals across the United States, using a consistent, harmonized protocol. Studies may build upon the NKI-RS andNoble et al. (2017)by collecting measurements from diverse groups across multiple neuroimaging sites or protocols. Our work provides a theoretical foundation for evaluating studies such as (Noble et al., 2017) and (Yamashita et al., 2019), which advocate aggregating multi-site*traveling subject*datasets to explore batch effects with minimal assumptions. This allows appreciation of internally valid demographic-specific effects and informs modeling assumptions, potentially enabling extrapolatory batch effect removal techniques in non-demographic-aligned datasets. Recent work highlights that data pre-processing strategies significantly impact subsequent derivatives (Bhagwat et al., 2021;Bridgeford et al., 2021;Gargouri et al., 2018;Vergara et al., 2017). While some strategies may optimally satisfy certain criteria (e.g., registration quality), they may improve or worsen batch effects. Future research could explore optimizing pre-processing to limit batch effects while meeting other requirements.

## Supplementary Material

Supplementary Material

## Data Availability

The raw data analyzed in this manuscript can be obtained at CoRR Mega-Study. The pre-processed data analyzed in this manuscript are available athttp://neurodata.io/mri. The methods introduced in this work are made accessible for users via the R package causalBatch (Bridgeford et al., 2024) (available on CRAN), which features numerous package vignettes to facilitate ease of use. Code for reproducing the figures and analyses contained in this work is available fromhttps://github.com/neurodata/causal_batch, and a docker container with all software versions for reproducing these results is available athttps://hub.docker.com/r/neurodata/causal_batch.
