# Supplementary material for: When no answer is better than a wrong answer: A causal perspective on batch effects
Source: Imaging Neurosci (Camb). 2025 Jan 29;3:imag_a_00458. doi: 10.1162/imag_a_00458 (PMC12319767; doi:10.1162/imag_a_00458)
Supplement: Supplementary Material [file imag_a_00458-supp.pdf]

Supplementary material for: When no answer is better than a wrong answer: a causal perspective on batch effects

Eric W. Bridgeford[1,2,†], Michael Powell[1], Gregory Kiar[3], Stephanie Noble[4], Jaewon Chung[1], Sambit Panda[1], Ross Lawrence[1], Ting Xu[3], Michael Milham[3], Brian Caffo[1], Joshua T. Vogelstein[1]

[1] Johns Hopkins University,

[2] Stanford University,

[3] Child Mind Institute,

[4] Yale University, Northeastern University,

[†]Correspondence: ericwb@stanford.edu

January 17, 2025

A Definitions

For a formal description of the below definitions, see Bridgeford et al. (2023). Informally, we adopt the following notation to describe batch effect correction. We assume that Y_i represents an observed measurement of interest (e.g., a connectome), T_i represents the batch in which the measurement is collected, X_i represents observed covariates, (e.g., age or biological sex), and Z_i represents unobserved covariates (e.g., height). The data (Y_i, T_i, X_i, Z_i) are n independent and identical samples from some true and unknown data generating distribution. The quantity $Y_i^{(t)}$ represents the measurement that would have been observed, had the measurement been collected in a given batch t. The key distinction is that Y_i represents the *actual* measurement, which is collected and studied. On the other hand, $Y_i^{(t)}$ is a potentially hypothetical measurement, which is only actually observed for the case where $T_i = t$ in most experimental contexts (Cole & Frangakis, 2009). That is, individuals have a potential measurement $Y_i^{(t)}$ for every possible batch, but only a single of these potential measurements are studied under standard observational contexts. This is known as the *consistency assumption*, and can be written mathematically as:

$$Y_i = \sum_{t \in \mathcal{T}} \mathbb{1}_{\{T_i = t\}} Y_i^{(t)} = Y_i^{(T_i)}, \tag{1}$$

where $\mathcal{T}$ is the set of batch labels. That the observed measurements Y_i given the batch (or measured covariates) and the potential measurements $Y_i^{(t)}$ do not, in general, have similar distributions is known as the "fundamental problem of causal inference." This problem requires resorting to additional assumptions to make conclusions on the basis of the observed data.

Using this convention, a batch effect is defined in Definition 1.

Definition 1 (Batch Effect)**.** *A batch effect exists between two batches t and t' if $Y_i^{(t)}$ and $Y_i^{(t')}$ have different distributions.*

In light of this definition, a batch effect can be conceptualized as the *potential* measurements having different distributions between a given pair of batches. A batch effect is present if an individual's potential measurements (which are random quantities) would differ (in terms of their distributions) *by virtue of them being measured* in batches t and t'.

The major conceptual leap is that, under the manner in which most mega-studies are collected (where individuals are *unique* to each batch), Definition 1 is about *potential* measurements rather than *realized* measurements. Causal claims regarding potential measurements will only be valid on the basis of the observed (realized) data insofar as the reasonableness of the assumptions upon which they rest.

A.1 Associational Effects

In an associational context, we observe measurements y_i and batches t_i for each individual $i \in [n]$, so effects can only be determined from realizations of Y_i and T_i.

Definition 2 (Associational Effect). *An associational effect exists between batches t and t' if $Y_i|T_i = t$ and $Y_i|T_i = t'$ have different distributions.*

Likewise, we can use this intuition to develop a suitable definition for associational batch effect correction:

Definition 3 (Associational Effect Correction). *Associational effect correction is a function g, where:*

$$g\left(\{(Y_i, T_i)\}_{i\in[n]}\right) = \left\{\tilde{Y}_i\right\}_{i\in[n]}$$

such that for all pairs of batches t and t', $\tilde{Y}_i|T_i = t$ and $\tilde{Y}_i|T_i = t'$ have the same distribution.

Whereas Y_i represents the measurement for an individual, the intuition is that $\tilde{Y}_i$ can be thought of as the "corrected" measurement, which does not have an associational effect for any pair of batches in the observed data.

Associational effects will often poorly characterize whether a batch effect is present. Consider, for instance, if in one batch t, we tend to measure older individuals, whereas in another batch t', we tend to measure younger individuals. If age is related to the measurement we obtained, then the differences between $Y_i|T_i = t$ and $Y_i|T_i = t'$ could be due to age or batch identity, and we have no way of differentiating whether the effect is a *bona fide* batch effect versus merely an associational effect. A sufficient condition for an associational effect to facilitate detecting or estimating a batch effect would be that individuals are randomized to each batch, in that individuals are randomly assigned to be measured in particular batches *pre-hoc*. Associational effect detection can be facilitated via Dcorr (Székely et al., 2007), and associational effect correction can be facilitated via `ComBat` (Johnson et al., 2007).

A.2 Conditional Effects

In a conditional context, we observe measurements y_i, batches t_i, and covariates x_i for all individuals $i \in [n]$, so effects can be determined from realizations of Y_i, T_i, and X_i.

Definition 4 (Conditional Effect). *A conditional effect exists between batches t and t' if for some covariate x, $Y_i|T_i = t, X_i = x$ and $Y_i|T_i = t', X_i = x$ have different distributions.*

We can define conditional effect correction using this logic:

Definition 5 (Conditional effect correction). *Conditional effect correction is a function g, where:*

$$g\Big(\{(Y_i, T_i, X_i)\}_{i\in[n]}\Big) = \Big\{\tilde{Y}_i\Big\}_{i\in[n]}$$

such that for all pairs of batches t and t' and for all covariate levels x, $\tilde{Y}_i|T_i = t, X_i = x$ and $\tilde{Y}_i|T_i = t', X_i = x$ have the same distribution.

Conceptually, given the consistency assumption from Equation 1, a conditional effect is equivalent to a batch effect if two conditions hold:

1. the measured covariates overlap in distribution between the batches (propensity overlap), and
2. the measured covariates provide all of the information regarding the mechanism about how people ended up in one batch versus the other (strong ignorability).

The former condition denotes that both batches must contain similar groups of people (in terms of measured covariates), and the latter condition specifies that the measured covariates X_i tell us all of the information needed to "exchange" measurements from one batch to the other. Borrowing the preceding example, even if we observe more young people in a batch t, we must still observe *some* young people in the other batch t'. In this sense, the measured covariates contain the information needed to "de-confound" disparities that might be batch effects or veridical effects due to upstream covariates. Therefore, when we make subsequent comparisons, we do not need to guess what people with similar covariates would have looked like in the other batch, and vice versa.

In this fashion, our comparisons can be thought of as locally (with respect to the covariates) exchanging a realized measurement Y_i in batch t with a realized measurement Y_i in batch t', where both individuals have similar covariates x. Intuitively, these comparisons can therefore be conceptualized as synthetically comparing $Y_i^{(t)}$ and $Y_i^{(t')}$ (the target estimand for establishing a batch effect) by using observed measurements Y_i from individuals who are similar on the basis of the covariates X_i between the two batches.

Condition 1 ensures that we can make this intuitive step over the entire span of covariates in our batches. Conditional effect detection can be facilitated via cDcorr (Wang et al., 2015), and conditional effect correction can be facilitated via cComBat (Johnson et al., 2007).

In practice, we never know whether the propensity distributions overlap; we can only estimate them from the data. If our estimated propensities do not overlap given finite data, we again cannot reasonably differentiate between differences in the two groups being due to *bona fide* batch effects or empirical differences in the propensity distributions. This motivates a third approach.

A.3 Adjusted Effects

As before, we observe measurements y_i, batches t_i, and covariates x_i for all individuals $i \in [n]$, and we determine effects from realizations of Y_i, T_i, and X_i. Prior to assessing the equality of any distributions, however, we weight the observations such that the observed covariate distributions are rendered approximately overlapping.

Definition 6 (Adjusted Effect)**.** *An adjusted effect exists between batches t and t' if after re-weighting samples such that $X_i|T_i = t$ and $X_i|T_i = t'$ are approximately overlapping (or, alternatively, approximately equal) in distribution, $Y_i|T_i = t, X_i = x$ and $Y_i|T_i = t', X_i = x$ have different distributions.*

We can similarly define adjusted effect correction as:

Definition 7 (Adjusted Effect Correction)**.** *Assume that the samples are re-weighted via weights w_i (possibly 0 or 1) such that after re-weighting, $X_i|T_i = t$ and $X_i = t'$ are approximately overlapping (or alternatively, approximately equal) in distribution for all $w_i \neq 0$.*

Adjusted effect correction is a function g, where:

$$g\Big(\{(Y_i, T_i, X_i, w_i)\}_{i\in[n]}\Big) = \Big\{\tilde{Y}_i\Big\}_{i\in[n]}$$

such that for all pairs of batches t and t' and for all covariate levels x, $\tilde{Y}_i|T_i = t, X_i = x$ and $\tilde{Y}_i|T_i = t', X_i = x$ have the same distribution.

Adjusted effects, by default, satisfy the first criterion for a conditional effect to be a batch effect. This is because rendering measured covariate distributions approximately equal intuitively is a more strict criterion than simply ensuring that they approximately overlap. The reason that we believe that re-weighting to ensure approximate covariate distribution equality is desirable for effect correction, versus simply approximate covariate overlap, is discussed in Section 3.1 and Supplementary Material D.3. Adjusted effect detection can be facilitated via `Causal cDcorr` (Bridgeford et al., 2023), and adjusted effect correction can be facilitated with `Matching cComBat` (described in Section 2.3).

We still must satisfy the latter criterion for a conditional effect to be a batch effect; that is, given the measured covariates, we can ignore how people ended up in one batch versus the other. This assumption has the same interpretation as before.

A.4 Crossover Effects

We observe measurements $y_i^{(t)}$ and covariates $x_i^{(t)}$, for all individuals $i \in [n]$ and for all batches $t \in \mathcal{T}$. In this case, we can make statements on the basis of $Y_i^{(t)}$ itself, because we actually observe outcomes for each possible batch. Therefore, we typically will not need to resort to local exchangeability (similar individuals/covariates across batches) as before, unless there are aspects of the individuals changing from one batch to another. *Crossover effects in general require the fewest assumptions to derive causal conclusions, since we directly observe all possible potential measurements for each individual.*

Definition 8 (Crossover Effect, constant states)**.** *A crossover effect exists between batches t and t' if, given that $\left(X_i^{(t)}, Z_i^{(t)}\right)$ and $\left(X_i^{(t')}, Z_i^{(t')}\right)$ are sufficiently similar, $Y_i^{(t)}$ and $Y_i^{(t')}$ have the same distribution.*

We are certain that any *traits* of the participants (i.e., variables that are constant for a given participant, such as genome) are the same across the two groups since the group members are identical (even if we did not measure these traits). However, *states* may differ as they may be functions of location or time. For example, if being measured impacts subsequent states, then a crossover effect may not be indicative of a batch effect without further assumptions and/or experimental design considerations (such as randomizing exposure order across participants, or resorting to adjusted effect strategies as before if these states are measured).

In the case where participant states are unchanging or are randomized, new associational strategies would need to be developed which, rather than comparing data directly across batches, batch effects would be

estimated (or detected) by looking at disparities that arise across batches for the same individual measured multiple times. For instance, instead of investigating batch effects by comparing across batches, one could investigate batch effects by instead looking at within-individual differences across batches, and then investigating batch effects by aggregating across these within-individual differences.

In the case where participant states are changing and are not randomized but are measured, we can resort to adjusted strategies for adjusted effect detection or correction, via a crossover effect for non-constant states:

Definition 9 (Crossover Effect, non-constant states)**.** *A crossover effect exists between batches t and t' if, after re-weighting samples such that $X_i^{(t)}$ and $X_i^{(t')}$ are approximately overlapping (or, alternatively, approximately equal) in distribution, $Y_i^{(t)}$ and $Y_i^{(t')}$ have the same distribution.*

For this effect to be a true batch effect, we need to make the same assumptions as before; that is, that given the measured covariates (which include the changing states), we can ignore how people ended up in one batch versus the other. New methods would need to be devised which combine covariate adjustment strategies with similar strategies to those proposed to address crossover effects with constant states.

B Statistical Methods

B.1 Hypothesis Testing

Recall that statements of the form $f(y) = g(y)$ against $f(y) \neq g(y)$ are equivalent to $P_f = P_g$ against $P_f \neq P_g$, as probability densities uniquely define distribution functions. Therefore, hypotheses for the effects described in Section 2.2 require two-sample and conditional two-sample testing procedures. A natural test statistic for the two-sample testing procedure is the Distance Correlation (Székely et al., 2007), which is a non-parametric test for testing whether two variables are correlated. A simple augmentation of the distance correlation procedure (Shen et al., 2017; Vogelstein et al., 2019) shows that `DCorr` can be used for the two-sample test, or a test of whether two samples are drawn from different distributions. `DCorr` is exactly equivalent in this context to the Maximum Mean Discrepency (MMD), which embeds points in a reproducing kernel Hilbert Space (RKHS) and looks for functions over the unit ball in the RKHS that maximize the difference of the means of the embedded points. When we instead consider the conditional two-sample test (i.e., a test of $f(y|x) = g(y|x)$ against $f(y|x) \neq g(y|x)$), we instead use the conditional distance correlation (Wang et al., 2015), a kernel-based approach in which the points are embedded in a new, non-linear Hilbert Space, which augments the traditional linear Hilbert Space used in distance correlation to allow the definition of the squared distance covariance. Below, we let $\mathbf{Y} = (y_i) \in \mathcal{Y}^n$ denote realizations across both samples and $\vec{t} = (t_i) \in \{t, t'\}^n$ indicate from which sample each realization is drawn. $\mathbf{X} = (x_i) \in \mathcal{X}^n$ denotes covariates which are known about the objects of interest.

Associational Effect We have the following null and alternative hypotheses:

$$H_0 : f(y|t) = f(y|t') \text{ against } H_A : f(y|t) \neq f(y|t')$$

A test of the preceding hypotheses is performed using the distance correlation, and the natural test statistic is $\texttt{DCorr}(\mathbf{Y}, \vec{t})$.

Conditional Effect We have the following null and alternative hypotheses:

$$H_0 : f(y|t, x) = f(y|t', x) \text{ against } H_A : f(y|t, x) \neq f(y|t', x)$$

A test of the preceding hypotheses is performed using the conditional distance correlation, and the natural test statistic is $\texttt{cDCorr}(\mathbf{Y}, \vec{t}|\mathbf{X})$.

Adjusted Conditional Effect We have the following null and alternative hypotheses:

$$H_0 : \tilde{f}(y|t,x) = \tilde{f}(y|t',x) \text{ against } H_A : \tilde{f}(y|t,x) \neq \tilde{f}(y|t',x)$$

Unlike the preceding tests, we instead consider the data $(\tilde{\mathbf{Y}}, \tilde{\vec{t}}, \tilde{\mathbf{X}})$, which are the measurements, sample indicators, and covariates of the n realizations after covariate adjustment. A test of the preceding hypotheses is performed using the conditional distance correlation, and the natural test statistic is $\texttt{cDCorr}(\tilde{\mathbf{Y}}, \tilde{\vec{t}}|\tilde{\mathbf{X}})$.

Causal Crossover Effect We have the following null and alternative hypotheses:

$$H_0 : f\left(y^{(t)}\middle|t, x^{(t)}\right) = f\left(y^{(t')}\middle|t', x^{(t')}\right) \text{ against } H_A : H_0 : f\left(y^{(t)}\middle|t, x^{(t)}\right) \neq f\left(y^{(t')}\middle|t', x^{(t')}\right)$$

If the known covariates are identical between batches t and t', we test the preceding hypotheses using the distance correlation, and the natural test statistic is $\texttt{DCorr}(\mathbf{Y}, \vec{t})$. If the known covariates are not identical between batches t and t', we test the preceding hypotheses using the conditional distance correlation, and the natural test statistic is $\texttt{cDCorr}(\mathbf{Y}, \vec{t}|\mathbf{X})$. Note that to our knowledge, there are no energy statistical methods similar to other tests utilized herein that we are aware of for naturally paired data with multivariate responses that are non-parametric, so we use these tests as surrogates due to the lack of an alternative. These tests may afford robustness to certain types of model misspecification, at the expense of violations of independence assumptions across repeated samples. Therefore, the outcomes of such tests should be interpreted with caution.

More Than Two Batches The above approaches generalize sufficiently to K batches using K-sample testing approaches (Bridgeford et al., 2023). With f_k for $k \in [K]$ denoting the densities associated with K sites or batches, this motivates hypotheses of the form:

$$H_0 : f_k = f_l \text{ for all } k, l \text{ against } H_A : f_k \neq f_l \text{ for some } k \neq l,$$

which can be tested using the distance correlation or the conditional distance correlation as above, with the caveat that $\vec{t}$ becomes the matrix $\mathbf{T} = (t_{i,k}) \in \{0,1\}^{n \times K}$. Each entry $t_{ik} = 1$ if sample i is in batch k, and 0 otherwise. For the purposes of this manuscript, we focus on the two-batch case.

B.2 Control Numerical Experiments

Control experiments are performed to ensure that after batch effect correction, the resulting data maintains interpretability and utility for scientific inquiry. Even if the data is devoid of batch effects, it must still be useful for downstream inference. For our connectome data, we investigate the preservation of demographic effects after batch effect correction.

Demographic Effect Demographic effects are investigated across both the subset of connectomes upon which `Matching cComBat` is executed (the matched American Clique). We observe the tuple $(y_i(k,l), s_i, a_i, t_i)$ for $i \in [n]$, and $k, l \in [V]$, where $V = 116$ denotes the number of parcels in the Automated Anatomical Labelling (AAL) parcellation (N Tzourio-Mazoyer et al., 2002). We suppose that $Y(k,l)$ is the $[0,1]$-valued random variable denoting the weight of edge (k,l), S is the binary-valued random variable denoting the biological sex, A is the positive real-valued random variable denoting age, and T is the $[K]$-valued random variable denoting the batch. We let $\vec{y}(k,l) = (y_i(k,l)) \in [0,1]^n$ denote the realized edge weights, $\vec{s} = (s_i) \in \{0,1\}^n$ denote the realized biological sexes, $\vec{a} = (a_i) \in \mathbb{R}^n$ denote the realized biological ages, and $\vec{t} = (t_i) \in [K]^n$ denote the realized batches. We say that a **demographic sex effect** exists when:

$$f(y|a,s) \neq f(y|a,s')$$

To test for a demographic sex effect, we have the following null and alternative hypotheses:

$$H_0 : f(y|a,s) = f(y|a,s') \text{ against } f(y|a,s) \neq f(y|a,s')$$

We are able to test the preceding hypothesis using the generalized covariance measure (Shah & Peters, 2018), and the test statistic is $\texttt{gcm}(\vec{y}(k,l), \vec{s}|\vec{a})$.

B.3 p-values and Multiple Hypothesis Correction

p-values in this manuscript are estimated using permutation testing, which is an approach to obtain the distribution of the test statistic under the null with minimal assumptions and approximations (Efron, 2004). All p-values are estimated using $R = 1{,}000$ (detection, Figure 6, and correction, Figure 7) permutations. Across all figures associated with this work, we are concerned with obtaining a proper estimate of the rate at which we detect effects (*discoveries*). Therefore, we control the false discovery rate (FDR) with the Benjamini-Hochberg Correction (Benjamini & Hochberg, 1995).

C Procedures for detecting and mitigating batch effects

C.1 Detecting batch effects with Causal cDcorr

Many of the more direct types of detectable effects, such as associational and conditional effects, fail to adaequately account for confounding biases present in the data. We instead propose the use of Causal cDcorr, in which a conditional K-sample test (Wang et al., 2015) is performed on samples with the same "range" of covariate values after propensity trimming via a strategy known as vector matching (Lopez & Gutman, 2017). Specifically, from (Bridgeford et al., 2023), given a dataset with batch assignments t_i, covariates x_i, and measurements y_i, Causal cDcorr is performed as follows:

1. Perform vertex matching, using the batch assignments given the covariate variables.
   (a) Perform a multinomial regression, regressing the batch assignments t_i onto the covariates x_i, to estimate a probability vector $\hat{r}(t, x_i)$ for each of the individuals for all batches $t \in [K]$.
   (b) For each batch t, use the procedure of (Lopez & Gutman, 2017) to produce high and low probability thresholds $l^{(t)}, h^{(t)}$.
   (c) Exclude samples j for which $\hat{r}(t, x_j) \notin (l^{(t)}, h^{(t)})$ for any $t \in [K]$. This step excludes samples which are overly probable (or improbable) to be from any particular batch.
2. One-hot-encode the batch assignments t_i to obtain the K-dimensional vectors $\vec{v}_i$, where $v_{it} = 1$ when $t = t_i$ and 0 otherwise.
3. Compute the distance correlation between y_i and $\vec{v}_i$ conditional on x_i, using cDcorr (Wang et al., 2015).

This strategy is the focus of a complementary theoretical manuscript, in which we illustrate the theoretical and empirical (via simulations) benefits of this technique over competing approaches for detecting causal effects between potential outcomes. This strategy maintains both high sensitivity and specificity under traditionally problematic data regimes (high-dimensionality, non-monotonicities, and non-linearities) in which other methods typically fail (Bridgeford et al., 2023), making it a natural choice for causal discrepancy testing (of which "batch effect" detection, termed *causal unconditional discrepancy testing* in (Bridgeford et al., 2023), as-defined herein is a special case).

Figure 5C illustrates visually the causal procedures employed for adjusting the batches. Rather than fully matching to produce the adjusted batches, samples are retained only such that they have approximately

overlapping covariate distributions (propensity trimming, shaded boxes). A full schematic illustrating the use of vector matching is detailed in Supplementary Material E of (Bridgeford et al., 2023). In the event that the batch assignment mechanism is ignorable given the covariates and that effects between datasets are in the same direction across all covariate levels, the adjusted effect detected by Causal cDcorr is a causal batch effect, as proven in (Bridgeford et al., 2023). That the effects between datasets are in the same "direction" across all covariate levels can be best intuited via example. Consider the case where a batch effect exists, such that there is a signal disparity (a difference in the expected connectivity) in a particular edge of a connectome between two batches. If this signal disparity is a positive difference between batches 1 and 2 across all covariate levels (or, a negative difference across all covariate levels), Causal cDcorr will detect a causal batch effect. In the event that the assignment mechanism is ignorable but that effects between datasets are not in the same direction across all covariate levels, the adjusted effect detected by Causal cDcorr is a causal effect, but may reflect a *causal conditional discrepancy* (e.g., there are batch-specific differences, but they are isolated to particular covariate levels). In the event that the batch assignment mechanism is not ignorable, the effect may not reflect any causal effects (e.g., it may reflect unmeasured demographic differences between the batches).

C.2 Mitigating batch effects using `Matching cComBat`

Unfortunately, many existing techniques for the removal of batch effects fail to adequately account for confounding biases that may be present in the data. We propose `Matching cComBat`, in which `cComBat` is performed on a subset of observational studies in which all pairs of studies are balanced against measured demographic covariates. `Matching cComBat` is performed as follows. Given measurements and covariates from n individuals across K batches, each of which has n_k individuals:

1. Perform vertex matching, using the batch assignments given the covariate variables.
2. Match control datasets to a reference dataset (defaults to smallest dataset).
   (a) Perform nearest neighbor matching (without replacement) for all pairs of control datasets against the reference. This matching is performed many-to-one or one-to-many, with the aim of retaining the maximum number of possible matched pairs. When possible, use exact matching for categorical and binary covariates, and Mahalanobis distance matching for continuous and ordinal covariates. Default behavior uses a 0.1-width distance caliper (maximum possible distance for continuous and ordinal covariates).
   (b) Retain all reference samples with a match to a control sample, and exclude all reference samples with no suitable matches (the matched reference samples).

   (c) Retain control samples matched to a reference sample, and exclude control samples which are unmatched (the matched control samples).

3. Perform `cComBat` (Johnson et al., 2007) on the measurements of the matched reference and matched control individuals across the K batches, conditioned on the measured covariates x_i.

In the event that the conditioning set closes backdoor paths (Pearl, 2009, 2010), `Matching cComBat` yields the removal of an internally valid causal effect and does not require extrapolation assumptions, unlike `cComBat` (Ho et al., 2011; Rosenbaum & Rubin, 1983, 1985; Stuart, 2010). If the conditioning set does not close backdoor paths, the effect removed is a conditional effect and may potentially yield the removal of demographic effects, as we saw in Figure 4. Supplementary Material E.2 depicts the impact on the empirical covariate distribution of the adjustment procedure.

C.3 Mitigating batch effects using `AIPW cComBat`

In addition to `Matching cComBat`, we propose `AIPW cComBat`, in which `cComBat` is combined with IPW methods to mitigate batch effects. `AIPW cComBat` is performed as follows. Given measurements and covariates from n individuals across K batches, each of which has n_k individuals, and a reference batch:

1. Perform vertex matching using the batch assignments given the covariate variables to remove samples with no overlap in covariate space.

2. Estimate propensity scores using multinomial logistic regression on the batch assignments given the covariates.

3. For each feature/dimension:

   (a) Fit separate outcome regression models for each batch using the specified covariate model, and

   (b) Calculate potential outcomes for each sample under each possible batch assignment using these models.

4. Compute the AIPW estimator for each batch and feature by:

   (a) Weighting the difference between observed and modeled outcomes by inverse propensity scores, and

   (b) Adding back the average modeled potential outcomes.

5. Adjust measurements by removing the estimated batch-specific component:

(a) Subtract the modeled outcome for the observed batch.

(b) Add back the modeled outcome for the reference batch.

This model provides double robustness, in that if either the outcome model for each batch or the propensity model are correctly specified, estimated batch effects are consistent for the true underlying batch effect, with respect to the reference batch.

C.4 Covariate Adjustment

The exposed group t defaults to be the smaller of the two groups, and the unexposed group t' is selected to be the larger of the two groups, where n_t is the number of individuals in the exposed group and $n_{t'}$ is the number of individuals in the unexposed group. The "covariate overlap" procedure attempts to ensure positivity of the propensity distribution for the unexposed group (i.e., $e(t'|x) > 0$). Intuitively, we exclude individuals from the unexposed group who do not appear "similar" to individuals in the exposed group. In this work, covariate overlap is established via vewrtex matching (Lopez & Gutman, 2017). Similarly, the "covariate balancing" procedure attempts to re-weight observations in the per-batch covariate distributions such that the covariate distributions are approximately equal (i.e., $f(x|t) \approx f(x|t')$). In this work, we perform $k : 1$ or $1 : k$ nearest-neighbor matching using the `MatchIt` package (Ho et al., 2011). The number of matches $k = \lfloor \frac{n_{t'}}{n_t} \rfloor$ is chosen to be the largest number of unexposed matches possible. Individuals are balanced on the basis of individual sex, individual age, and continent of study. As there are likely many other categories with which brain connectivity may be confounded, we do not believe this covariate set is necessarily sufficient to identify a Causal Batch Effect (Definition 1), as we would need to be confident that these covariates exhibited the covariate sufficiency property. We perform exact matching on the basis of individual sex and individual continent of measurement, and use a 0.1-width distance caliper on the propensity score to obtain at most k matched participants for each treated individual (Powell et al., 2020).

D Simulations

D.1 Batch Effect Detection Simulations

Simulations illustrating the sensitivity (high testing power when a causal effect is present) and specificity (tests which do not falsely detect effects) of Causal cDcorr for causal effect detection are in Bridgeford et al. (2023).

D.2 Simple Simulations

This delineates the simulation settings for Figure 1 in our manuscript.

$n = 500$ points are sampled from Batch 0 or Batch 1 with probability 0.5; e.g., $T_i \overset{iid}{\sim} \text{Bern}(0.5)$.

D.2.1 Covariate distributions

In Figure 1, the covariate distributions are determined by:

$$X_i | T_i = t \overset{d}{\sim} \begin{cases} 2\text{Beta}(2, 4) - 1 & t = 0 \\ 2\text{Beta}(4, 2) - 1 & t = 1 \end{cases}$$

D.2.2 Outcome model

With $\epsilon_i \overset{iid}{\sim} \mathcal{N}(0, 1)$, and the batch effect β is either 1 (bottom row) or -1 (top row):

$$Y_i = -4\text{sigmoid}(8X_i) + 4 + \beta T_i + \frac{1}{2}\epsilon_i,$$

D.3 Full Simulations

This delineates the simulation settings for Figure 4 in our manuscript.

$n = 1000$ points are sampled from Batch 0 or Batch 1 with probability 0.5; e.g. $T_i \overset{iid}{\sim} \text{Bern}(0.5)$.

D.3.1 Covariate distributions

In Figure 4, the covariate distributions are determined by:

$$X_i|T_i = t \overset{d}{\sim} \begin{cases} 2\text{Beta}(2, 2b) - 1 & t = 0 \\ 2\text{Beta}(2b, 2) - 1 & t = 1 \end{cases}$$

where b denotes the "unbalancedness". We vary b from 1 to 5. Letting f_0 denote the probability density function of $X_i|T_i = 0$ and f_1 the probability density function of $X_i|T_i = 1$, the covariate overlap is:

$$\text{overlap} = \int_{-1}^{1} \min\left(f_0(x), f_1(x)\right) \mathrm{d}x.$$

D.3.2 Simulation contexts

We investigate these in three simulation contexts, where for all simulations, $\epsilon_i \overset{iid}{\sim} \mathcal{N}(0, 1)$, and the batch effect β is either -1 (Batch Effect) or 0 (No Batch Effect):

Non-linearity A sigmoidal relationship between the covariate and the outcome. The outcome is:

$$Y_i = -4\text{sigmoid}(8X_i) + 4 + \beta T_i + \frac{1}{2}\epsilon_i,$$

where sigmoid(x) is the non-linear sigmoid function; e.g.:

$$\text{sigmoid}(x) = \frac{1}{1 + \exp(-x)}.$$

Non-monotonicity A gaussian non-monotonic relationship between the covariate and the outcome. The outcome is:

$$Y_i = 4\varphi\left(X_i, \mu = -0.5, \sigma = \frac{1}{2}\right) + \beta T_i + \frac{1}{2}\epsilon_i.$$

This non-monotonicity is "asymmetric" because $\mu = -0.5$, which leads to the effect not being symmetric about $x = 0$ (whereas the covariates are, by construction, symmetric about $x = 0$).

Linear A linear relationship between the covariate and the outcome. The outcome is:

$$Y_i = -2(X_i - 1) + \beta T_i + \frac{1}{2}\epsilon_i.$$

D.4 Estimated Absolute Average Treatment Effect

To evaluate the effectiveness of each batch effect correction technique on simulated data, we compute the true data expected signal for each covariate level; e.g., $\mathbb{E}\left[Y_i^{(t)}|x\right]$ for each batch t and each covariate level x. Since ϵ_i has mean 0, this would be the quantity:

$$\mathbb{E}\left[Y_i^{(t)}|x\right] = f(x) - t,$$

where f is the covariate/outcome relationship (possibly incorrectly modeled). Since x is continuous, we compute this value for x across $B = 200$ evenly spaced breakpoints for evaluation. Given a set of samples, we train a batch effect correction model, and fit the trained model to the expected signal for each covariate level, leaving us with corrected expected signal for each batch, which we denote by $v(t, x)$. In theory, if the batch effect correction technique removes the batch effect as modeled, $v(1, x) \approx v(0, x)$ for all x. To evaluate each technique, we consider the estimated average absolute treatment effect (estimated AATE, for brevity) for each trial r of 1000 trials:

$$d_r = \frac{1}{B}\sum_{x \in \mathcal{X}} \left|v(1, x) - v(0, x)\right|,$$

and the magnitudes of d_r are annotated in the plots (shaded red boxes) for a single simulation. We compute the mean estimated average absolute treatment effect (Mean Absolute ATE, Mean AATE) as:

$$\text{mean AATE} = \frac{1}{R}\sum_{r \in [R]} d_r.$$

A value of 0 corresponds to the batch effect being completely eliminated (the expected signal for each batch after correction is identical), a value of 1 would equate to the AATE between the expected signals being the same as before batch effect correction, and a value > 1 corresponds to the residual disparity between the expected signals *exceeding* the original batch effect. The procedure for simulations is illustrated in Figure 8.

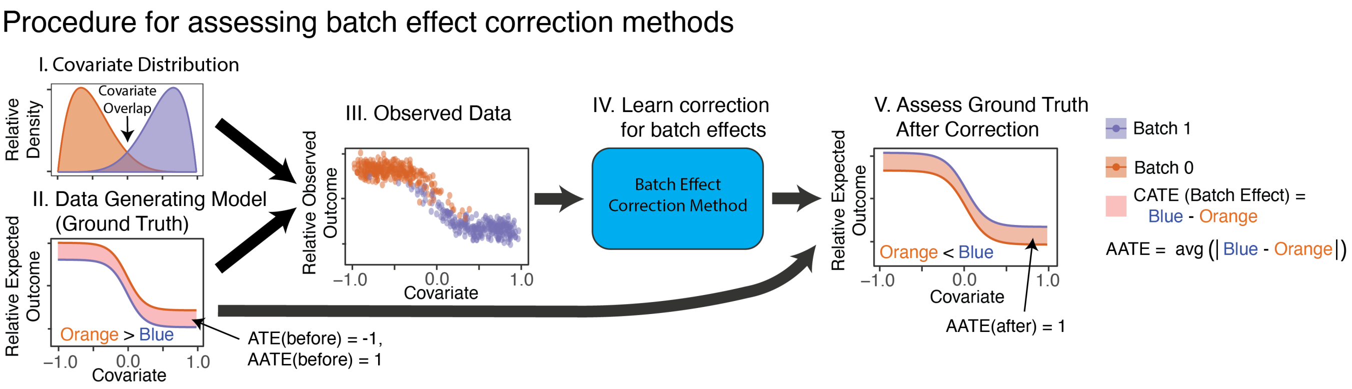

Figure 8: The procedure for assessing simulation performance in Figure 1. **I.** n individuals are sampled with equal probability from each batch. **II.** illustrates the generative model for the data in each batch. The ATE before correction is -1, and the average absolute treatment effect (AATE) is 1. **III.** illustrates the samples for each batch. **IV.** illustrates that the samples are used to train a batch effect correction model. **V.** the batch effect correction is applied to the data generating model, and the AATE is again computed between the two batches (and is still 1 here; e.g., the batch effect has not been removed). This procedure is repeated $R = 1000$ times for a given setting to produce the mean estimated AATE.

D.5 No batch effect simulations

Figure 9A shows similar simulations to Figure 4, but now there is no batch effect whatsoever (the AATE before correction is 0). The goal of these simulations is to identify whether the different methods are able to identify the lack of a batch effect, and avoid introducing a batch effect. All the methods correctly estimate that there is no batch effect in the linear setting (Figure 9B.I). However, non-causal methods incorrectly estimate the presence of a batch effect for both the nonlinear and non-monotonic settings, except when the covariates are nearly perfectly overlapping (Figure 9B.II and Figure 9B.III), and introduce batch effects to the data. In these regimes, non-causal techniques tend to introduce a batch effect, when none is present *a priori*. The causal methods behave better here, correctly identifying the relative absence of a batch effect (and therefore avoiding the introduction of a batch effect).

We evaluate how well the corrected data reflects the true underlying relationship (linear, non-linear, or non-monotonic) between the covariate and the outcome. We compare the corrected data to the true relationship using Pearson's correlation (Pearson, 1896), restricting to the matched samples so that the correlations are all computed with respect to the same set of sample in Figure 9(C). Low correlations indicate that the data poorly reflect the true relationship, suggesting that regardless of whether a batch effect were introduced to the data, the underlying signal has been perturbed. Causal methods here too tend to outperform non-causal methods for preserving the underlying signal in the data, and show performance near that of the `oracle`, particularly as covariate overlap declines.

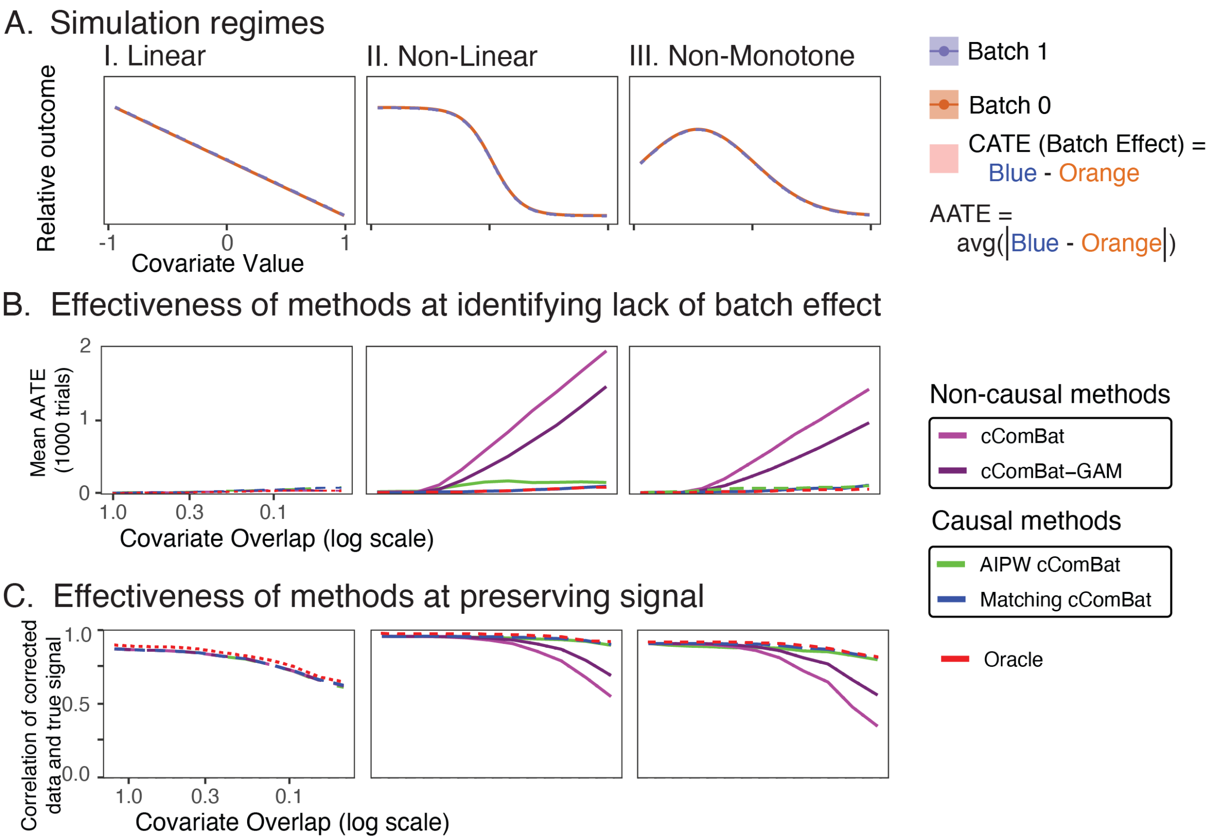

Figure 9: **Simulation regimes illustrate that non-causal procedures are subject to strong biases without covariate matching**. **(A)** illustrates the relationship between the relative expected outcome and the covariate value, for each batch (color), across **(I.)** linear, **(II.)** non-linear, and **(III.)** non-monotone regimes. The conditional average treatment effect (red box) highlights the batch effect for each covariate value. The average treatment effect (ATE) is the average width of this box, and the average absolute treatment effect (AATE) is the average absolute width of this box. In these simulations, the AATE before correction is 0. **(B)** The effectiveness of the techniques at avoiding the introduction of batch effects. Techniques with high performance will have a mean AATE after correction at or near 0 (no artifacts were introduced). **(C)** illustrates the effectiveness of different batch effect correction techniques for preserving the underlying true signal. Techniques with high performance will have higher correlations with the underlying true signal.

E Datasets

E.1 Consortium for Reliability and Reproducibiliy (CoRR) data Pre-processing

The CoRR Mega-Study (Zuo et al., 2014) is an aggregate dataset consisting of 27 studies collected with a similar goal: assessing the reliability and reproducibility of neuroimaging data. The mega-study consists of 1313 individuals, most of whom are measured numerous times, for a total of $N = 3597$ connectomes. All connectomes are estimated using the `m2g` (MRI to Graphs) pipeline (Kiar et al., 2018), which provides a wrapper for the `CPAC` Pipeline (Craddock et al., 2013). fMRI scans for each individual are first processed to remove motion artifacts using `mcflirt` (Jenkinson et al., 2002). The fMRI scans are then registered to the corresponding individual's anatomical scan using FSL's boundary-based registration (BBR) via `epireg` (Greve & Fischl, 2009). A non-linear transformation from the individual's anatomical scan to the MNI152 (Fonov et al., 2009) template is estimtaed using `FNIRT` (Jenkinson et al., 2012). Nuisance artifacts are removed by fitting the voxelwise timeseries to a regression model incorporating regressors for the Friston 24-parameter model (Friston et al., 1996), the top five principal components of the voxelwise timeseries in cerebrospinal fluid `aCompCor` (Behzadi et al., 2007), and a quadratic drift term. The adjusted voxelwise timeseries is downsampled to the regions of interest (ROIs) of the Automated Anatomical Labelling (AAL) parcellation (N Tzourio-Mazoyer et al., 2002) by taking the spatial mean signal for each timepoint across voxels within the region of interest. Functional connectivity is estimated using the pairwise correlation between all pairs of ROI timeseries within the AAL parcellation. Parcels are sorted throughout the manuscript according to hemispheric order, in which the parcels are aligned with left hemisphere parcels followed by right hemisphere parcels. Within hemisphere, parcels are sorted by AAL parcel number. For each study, we have baseline covariates for the continent, sex, and the age of participants.

The American Clique The "American Clique" describes a subset of the CoRR Mega-Study in which the sample populations share similarities in sample demographic characteristics. These studies share a demographic focusing on males and females (in roughly equal proportions) of individuals across a wide age range, and include the "NYU2", "IBATRT", "MRN1", "UWM", and "NYU1" studies. The 833 connectomes comprising the studies of the American clique are reduced to the $N = 284$ connectomes with maximal demographic overlap identified through covariate adjustment (described in Section C.4).

The NKI Rockland Sample (Nooner et al., 2012) is a single study from the CoRR Mega-Study consisting of 24 individuals, each of whom is measured two times across three functional MRI acquisition

protocols, which vary in the repetition time for each slice of the sequence (TR). The data was collected with the intention of investigating the impact of the different MRI protocols in a crossover-randomized approach. Due to the crossover property, evidence in favor of an effect provides strong evidence of a causal batch effect. Images with a TR of 645 millisecond (ms), 1400 ms, and 2500 ms are measured, with the prompt for each subject remaining identical.

E.2 Overlap of Empirical Covariates

The empirical overlap of the covariate distributions is difficult to compute in the case of data without making heavy parametric assumptions. For this reason, we turn to the distribution-free overlapping index (Pastore & Calcagnì, 2019). The distribution free overlapping index, $\hat{v}_{kl}$, is computed by first approximating the density of the distribution of the measured covariates for each dataset d, $X = (A, S, C)$, where A is a random variable whose realizations $a \in \mathcal{A}$ are ages, S is a random variable whose realizations $s \in \mathcal{S}$ are sexes (M or F), and C is a random variable whose realizations $c \in \mathcal{C}$ denote continent, using the base R function `stats::density`. The random variable D has realizations $d \in \mathcal{D}$ whose realizations denote dataset. The density $f_d(a|S = s, C = c)$ is the conditional density of age, conditional on the individual's sex being s, continent of measurement being c for a given dataset d. The mass $\mathbb{P}_d(S = s|C = c)$ is the conditional mass of sex, conditional on the individual's continent of measurement being c, for dataset d. Finally, the mass $\mathbb{P}_d(C = c)$ represents the mass of an individual's continent of measurement being c, for dataset d (0 or 1 for all d, since all individuals from dataset d are either measured on continent c or not). An estimate of the overlap between the two densities, $\hat{v}_{kl}$ between datasets k and l, is computed using the formula:

$$\hat{v}_{kl} = \sum_{c \in \mathcal{C}} \min\Big(\hat{\mathbb{P}}_k(C = c), \hat{\mathbb{P}}_l(C = c)\Big) \sum_{s \in \{\text{M,F}\}} \min\Big(\hat{\mathbb{P}}_k(S = s|C = c), \hat{\mathbb{P}}_l(S = s|C = c)\Big) \Bigg[\int_{\mathcal{A}} \min\Big(\hat{f}_d(a|S = s, C = c), \hat{f}_l(a|S = s, C = c)\Big) \,\mathrm{d}a \Bigg]$$

which is obtained via numerical quadrature.

Intuitively, this can be conceptualized as representing the mass of the "area under the curve" which is shared by the two densities for datasets k and l.

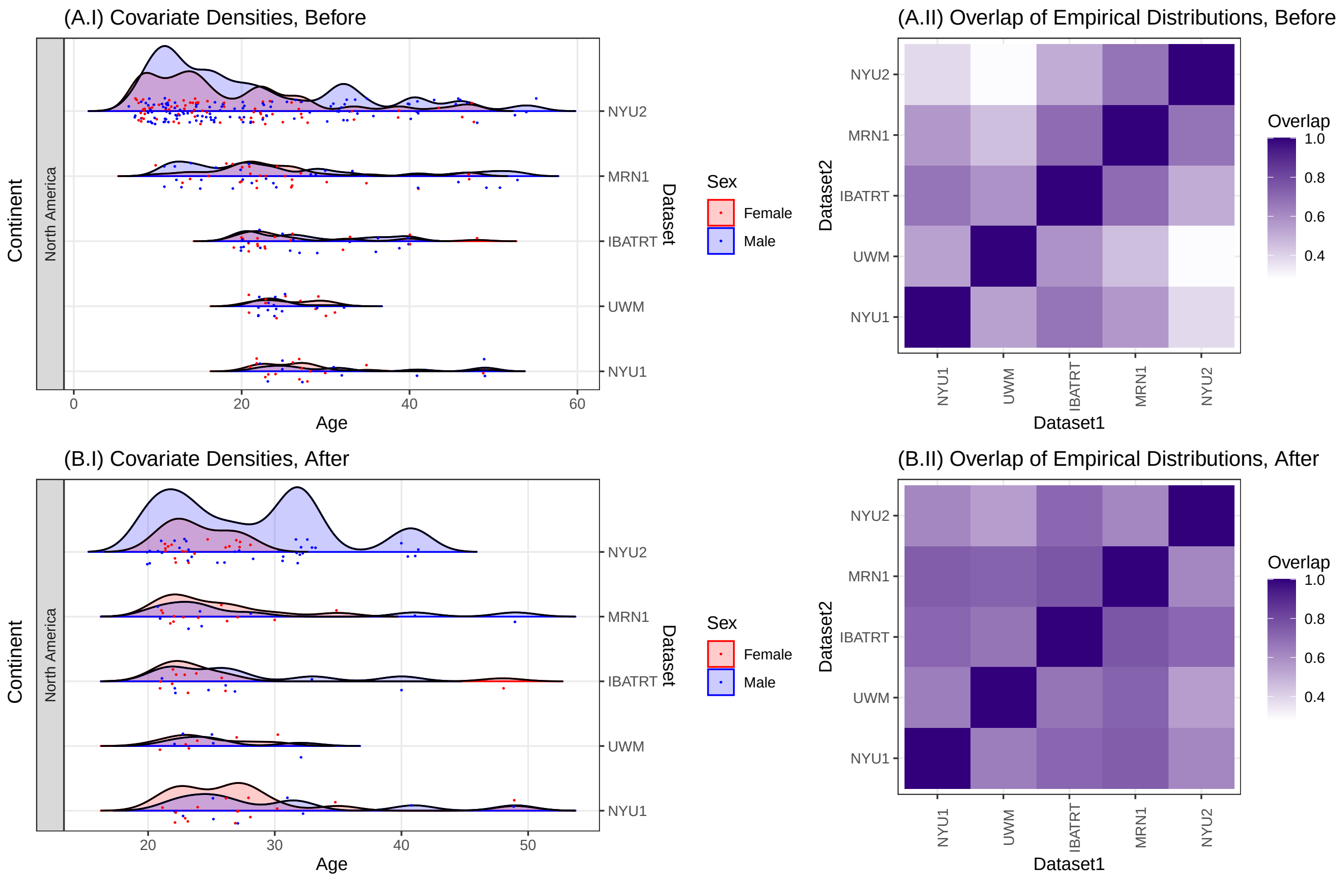

Figure 10: **The overlap of the empirical covariate distributions for the American Clique, before and after adjustment**. **(A)** The empirical distributions of the covariates before adjustment. **(B)** The empirical overlap of the covariate distributions after the adjustment procedure is applied, as discussed in Supplementary Material E.2.

E.3 Preservation of within-individual signal

The most fundamental properties of interest for bach effect correction methods to satisfy are that, for each individual, the connectomes after correction can be interpreted in the same context as the connectomes before correction. In this light, we investigate whether the topological properties of the connectomes are similar after correction as before. Figure 12(A) shows the connectomes before (Raw) and after batch effect correction is applied, by computing the cross-individual mean connectome. Note that before and after batch effect correction, the connectomes appear topologically similar, in that the relative edge-weights (across the methods) appear relatively consistent.

Figure 12(B) considers two properties of functional connectomes, homotopy and homophily. Homotopic edges are edges between ROIs in the same hemisphere of the brain (e.g., two an edge between two nodes

in the left hemisphere). Homophilic edges are edges between ROIs which denote the same brain area, but are in opposite hemispheres of the brain (e.g., an edge between the left and right motor cortex). In general, functional connectomes show a slight homophilic effect, and a very strong homotopic effect (Chung et al., 2020). We compute the effect size for each individual before and after correction, as the difference in average connectivity for edges in the noted edge group and edges not in the noted edge group (e.g., a comparison between the average connectivity of homophilic and non-homophilic edges, for "Homophilic", and a comparison between the average connectivity of homotopic and non-homotopic edges, for "Homotopic"). Points falling along the diagonal dotted black line $y = x$ tend to have a similar signal effect before and after batch correction, which includes the vast majority of the individuals.

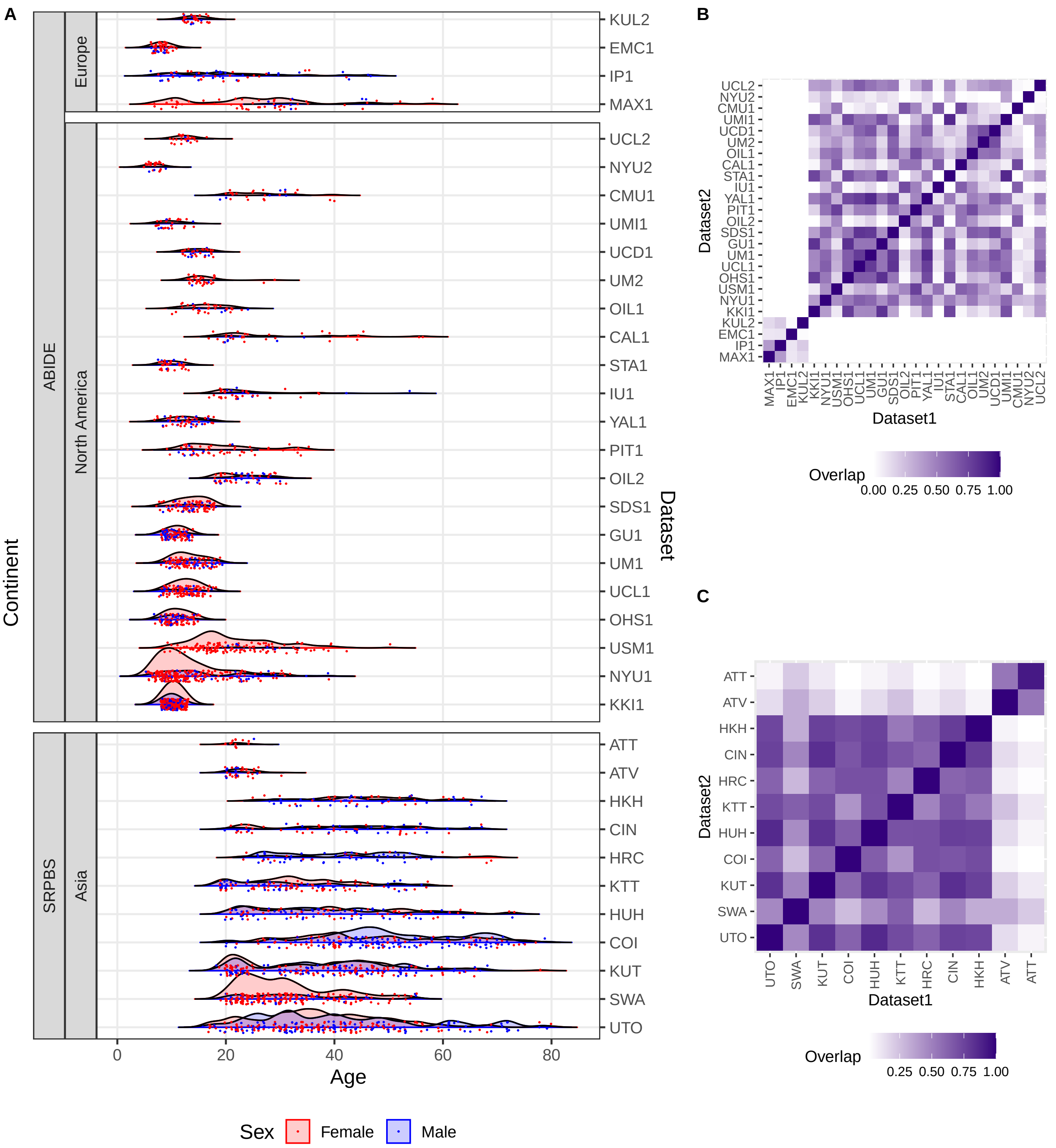

Figure 11: **The overlap of the empirical covariate distributions for two mega-studies. (A)** The empirical distribution of covariates. **(B)** The overlap of covariate distributions given by the distribution-free overlapping index for ABIDE mega-study (Di Martino et al., 2014, 2017). **(C)** The overlap of covariate distributions given by the distribution-free overlapping index for the SRPBS mega-study. (Yamashita et al., 2019). Like for the CoRR mega-study, while several pairs of sites have overlapping demographic distributions, many of the sites have extremely poor overlap in both mega-studies. In these cases, attempts to normalize for batch effects using model-based approaches like `cComBat` would be subject to the pitfalls of Figure 4 if modeling assumptions are not reasonable.

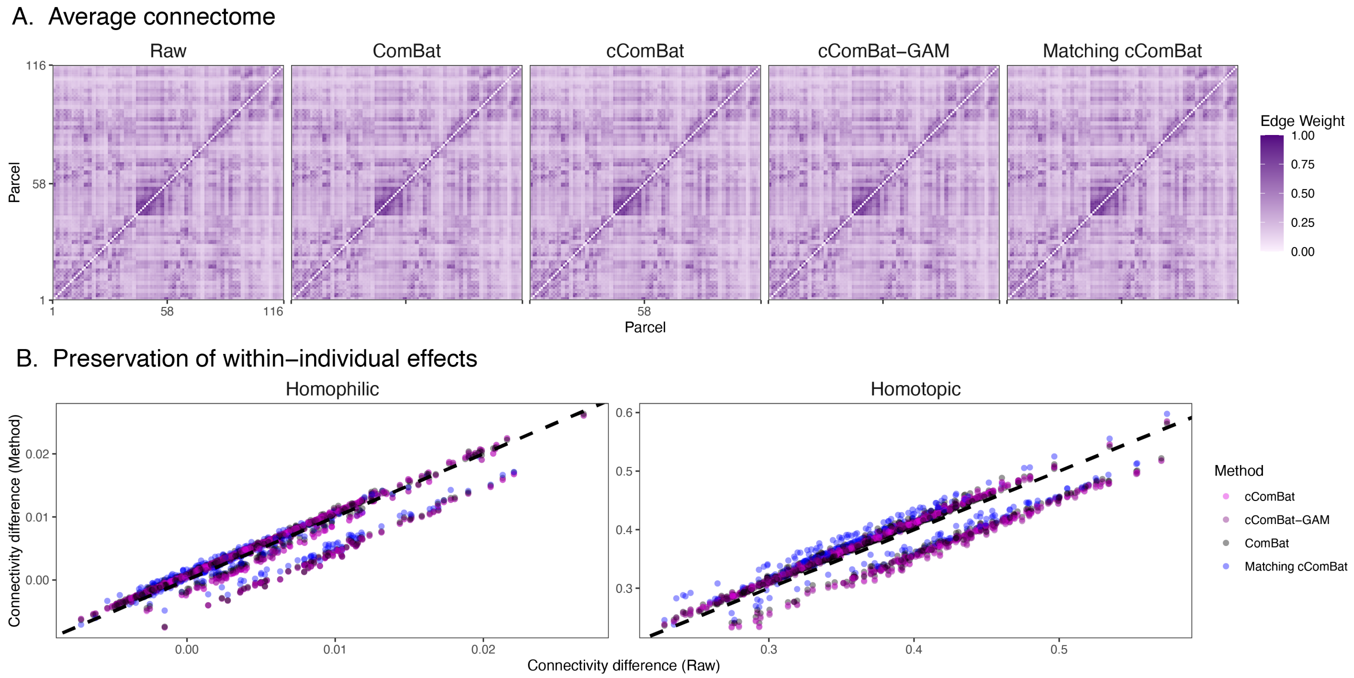

Figure 12: **Preservation of Topological Properties of Connectomes after Batch Correction**. **(A)** The average connectome, before (Raw) and after batch effect correction (other columns) across all individuals in the American Clique. **(B)** Scatter plots of two topological features of connectomes, homophily and homotopy. The *x-axis* denotes the average edge weight between edges which satisfy the noted feature and edges which do not, before any correction is applied (the "raw" connectomes). The *y-axis* denotes the same property for connectomes after correction is applied (point color).

References

Behzadi, Y., Restom, K., Liau, J., & Liu, T. T. (2007). A Component Based Noise Correction Method (CompCor) for BOLD and Perfusion Based fMRI. *Neuroimage*, *37*(1), 90. https://doi.org/10.1016/j.neuroimage.2007.04.042

Benjamini, Y., & Hochberg, Y. (1995). Controlling the False Discovery Rate: A Practical and Powerful Approach to Multiple Testing. *Journal of the Royal Statistical Society*.

Bridgeford, E. W., Chung, J., Gilbert, B., Panda, S., Li, A., Shen, C., Badea, A., Caffo, B., & Vogelstein, J. T. (2023). Learning sources of variability from high-dimensional observational studies. *ArXiv e-prints*. https://doi.org/10.48550/arXiv.2307.13868

Chung, J., Bridgeford, E., Arroyo, J., Pedigo, B. D., Saad-Eldin, A., Gopalakrishnan, V., Xiang, L., Priebe, C. E., & Vogelstein, J. T. (2020). Statistical Connectomics. *OSF Preprints*. https://doi.org/10.31219/osf.io/ek4n3

Cole, S. R., & Frangakis, C. E. (2009). The Consistency Statement in Causal Inference: A Definition or an Assumption? *Epidemiology*, *20*(1), 3. https://doi.org/10.1097/EDE.0b013e31818ef366

Craddock, C., Sharad, S., Brian, C., Ranjeet, K., Satrajit, G., Chaogan, Y., Qingyang, L., Daniel, L., Vogelstein, J., Burns, R., Stanley, C., Mennes, M., Clare, K., Adriana, D., Castellanos, F., & Michael, M. (2013). Towards Automated Analysis of Connectomes: The Configurable Pipeline for the Analysis of Connectomes (C-PAC). *Front. Neuroinf.*, *7*. https://doi.org/10.3389/conf.fninf.2013.09.00042

Di Martino, A., O'connor, D., Chen, B., Alaerts, K., Anderson, J. S., Assaf, M., Balsters, J. H., Baxter, L., Beggiato, A., Bernaerts, S., et al. (2017). Enhancing studies of the connectome in autism using the autism brain imaging data exchange ii. *Scientific data*, *4*(1), 1–15.

Di Martino, A., Yan, C.-G., Li, Q., Denio, E., Castellanos, F. X., Alaerts, K., Anderson, J. S., Assaf, M., Bookheimer, S. Y., Dapretto, M., et al. (2014). The autism brain imaging data exchange: Towards a large-scale evaluation of the intrinsic brain architecture in autism. *Molecular psychiatry*, *19*(6), 659–667.

Efron, B. (2004). Large-Scale Simultaneous Hypothesis Testing. *J. Am. Stat. Assoc.*, *99*(465), 96–104. https://doi.org/10.1198/016214504000000089

Fonov, V. S., Evans, A. C., McKinstry, R. C., Almli, C. R., & Collins, D. L. (2009). Unbiased nonlinear average age-appropriate brain templates from birth to adulthood. *Neuroimage*, *47*, S102. https://doi.org/10.1016/S1053-8119(09)70884-5

Friston, K. J., Williams, S., Howard, R., Frackowiak, R. S., & Turner, R. (1996). Movement-related effects in fMRI time-series. *Magn. Reson. Med.*, *35*(3), 346–355. https://doi.org/10.1002/mrm.1910350312

Greve, D. N., & Fischl, B. (2009). Accurate and robust brain image alignment using boundary-based registration. *Neuroimage*, *48*(1), 63–72. https://doi.org/10.1016/j.neuroimage.2009.06.060

Ho, D. E., Imai, K., King, G., & Stuart, E. A. (2011). MatchIt: Nonparametric preprocessing for parametric causal inference. *Journal of Statistical Software*, *42*(8), 1–28. https://www.jstatsoft.org/v42/i08/

Jenkinson, M., Bannister, P., Brady, M., & Smith, S. (2002). Improved optimization for the robust and accurate linear registration and motion correction of brain images. *Neuroimage*, *17*(2), 825–841. https://doi.org/10.1016/s1053-8119(02)91132-8

Jenkinson, M., Beckmann, C. F., Behrens, T. E. J., Woolrich, M. W., & Smith, S. M. (2012). FSL. *Neuroimage*, *62*(2), 782–790. https://doi.org/10.1016/j.neuroimage.2011.09.015

Johnson, W. E., Li, C., & Rabinovic, A. (2007). Adjusting batch effects in microarray expression data using empirical Bayes methods. *Biostatistics*, *8*(1), 118–127. https://doi.org/10.1093/biostatistics/kxj037

Kiar, G., Bridgeford, E. W., Gray Roncal, W. R., Chandrashekhar, V., Mhembere, D., Ryman, S., Zuo, X.-N., Margulies, D. S., Craddock, R. C., Priebe, C. E., Jung, R., Calhoun, V. D., Caffo, B., Burns, R., Milham, M. P., & Vogelstein, J. T. (2018). A High-Throughput Pipeline Identifies Robust Connectomes But Troublesome Variability. *bioRxiv*, 188706. https://doi.org/10.1101/188706

Lopez, M. J., & Gutman, R. (2017). Estimation of Causal Effects with Multiple Treatments: A Review and New Ideas. *Statist. Sci.*, *32*(3), 432–454. https://doi.org/10.1214/17-STS612

N Tzourio-Mazoyer, N., Landeau, B., Papathanassiou, D., Crivello, F., Etard, O., Delcroix, N., Mazoyer, B., & Joliot, M. (2002). Automated anatomical labeling of activations in SPM using a macroscopic anatomical parcellation of the MNI MRI single-subject brain. *Neuroimage*. https://doi.org/10.1006/nimg.2001.0978

Nooner, K. B., Colcombe, S., Tobe, R., Mennes, M., Benedict, M., Moreno, A., Panek, L., Brown, S., Zavitz, S., Li, Q., Sikka, S., Gutman, D., Bangaru, S., Schlachter, R. T., Kamiel, S., Anwar, A., Hinz, C., Kaplan, M., Rachlin, A., . . . Milham, M. (2012). The NKI-Rockland Sample: A Model for Accelerating the Pace of Discovery Science in Psychiatry. *Front. Neurosci.*, *6*. https://doi.org/10.3389/fnins.2012.00152

Pastore, M., & Calcagnì, A. (2019). Measuring Distribution Similarities Between Samples: A Distribution-Free Overlapping Index. *Front. Psychol.*, *0*. https://doi.org/10.3389/fpsyg.2019.01089

Pearl, J. (2009). Causal inference in statistics: An overview. *ssu*, *3*(none), 96–146. https://doi.org/10.1214/09-SS057

Pearl, J. (2010, July). *On measurement bias in causal inference*. AUAI Press. https://doi.org/10.5555/3023549.3023599

Pearson, K. (1896). Mathematical contributions to the theory of evolution. iii. regression, heredity, and panmixia. *Philosophical Transactions of the Royal Society A: Mathematical, Physical and Engineering Sciences*, *187*, 253–318.

Powell, M., Koenecke, A., Byrd, J. B., Nishimura, A., Konig, M. F., Xiong, R., Mahmood, S., Mucaj, V., Bettegowda, C., Rose, L., Tamang, S., Sacarny, A., Caffo, B., Athey, S., Stuart, E. A., & Vogelstein, J. T. (2020). A how-to guide for conducting retrospective analyses: example COVID-19 study. *OSF Preprints*. https://doi.org/10.31219/osf.io/3drch

Rosenbaum, P. R., & Rubin, D. B. (1983). The central role of the propensity score in observational studies for causal effects. *Biometrika*, *70*(1), 41–55. https://doi.org/10.1093/biomet/70.1.41

Rosenbaum, P. R., & Rubin, D. B. (1985). Constructing a control group using multivariate matched sampling methods that incorporate the propensity score. *The American Statistician*, *39*(1), 33–38. http://www.jstor.org/stable/2683903

Shah, R. D., & Peters, J. (2018). The hardness of conditional independence testing and the generalised covariance measure. *Annals of Statistics*. https://www.semanticscholar.org/paper/The-hardness-of-conditional-independence-testing-Shah-Peters/22c3262ac4d60531c452573c5e85cfbadb89df93

Shen, C., Priebe, C. E., & Vogelstein, J. T. (2017). From Distance Correlation to Multiscale Generalized Correlation. *Journal of American Statistical Association*. http://arxiv.org/abs/1710.09768

Stuart, E. A. (2010). Matching Methods for Causal Inference: A Review and a Look Forward. *Statist. Sci.*, *25*(1), 1–21. https://doi.org/10.1214/09-STS313

Székely, G. J., Rizzo, M. L., & Bakirov, N. K. (2007). Measuring and testing dependence by correlation of distances. *Ann. Stat.*, *35*(6), 2769–2794. https://doi.org/10.1214/009053607000000505

Vogelstein, J. T., Bridgeford, E. W., Wang, Q., Priebe, C. E., Maggioni, M., & Shen, C. (2019). Discovering and deciphering relationships across disparate data modalities. *Elife*, *8*. http://dx.doi.org/10.7554/eLife.41690

Wang, X., Pan, W., Hu, W., Tian, Y., & Zhang, H. (2015). Conditional Distance Correlation. *J. Am. Stat. Assoc.*, *110*(512), 1726. https://doi.org/10.1080/01621459.2014.993081

Yamashita, A., Yahata, N., Itahashi, T., Lisi, G., Yamada, T., Ichikawa, N., Takamura, M., Yoshihara, Y., Kunimatsu, A., Okada, N., Yamagata, H., Matsuo, K., Hashimoto, R., Okada, G., Sakai, Y., Morimoto, J., Narumoto, J., Shimada, Y., Kasai, K., . . . Imamizu, H. (2019). Harmonization of resting-state functional MRI data across multiple imaging sites via the separation of site differences into sampling bias and measurement bias. *PLoS Biol.*, *17*(4), e3000042. https://doi.org/10.1371/journal.pbio.3000042

Zuo, X.-N., Anderson, J. S., Bellec, P., Birn, R. M., Biswal, B. B., Blautzik, J., Breitner, J. C. S., Buckner, R. L., Calhoun, V. D., Castellanos, F. X., Chen, A., Chen, B., Chen, J., Chen, X., Colcombe, S. J., Courtney, W., Craddock, R. C., Di Martino, A., Dong, H.-M., . . . Milham,

M. P. (2014). An open science resource for establishing reliability and reproducibility in functional connectomics. *Sci. Data*, *1*(140049), 1–13. https://doi.org/10.1038/sdata.2014.49
